# A novel and universal method for microRNA RT-qPCR data normalization

**DOI:** 10.1186/gb-2009-10-6-r64

**Published:** 2009-06-16

**Authors:** Pieter Mestdagh, Pieter Van Vlierberghe, An De Weer, Daniel Muth, Frank Westermann, Frank Speleman, Jo Vandesompele

**Affiliations:** 1 Center for Medical Genetics, Ghent University Hospital, De Pintelaan 185, Ghent, Belgium; 2 Department of Tumour Genetics, German Cancer Center, Im Neuenheimer Feld 280, Heidelberg, Germany

## Abstract

Gene expression analysis of microRNA molecules is becoming increasingly important. In this study we assess the use of the mean expression value of all expressed microRNAs in a given sample as a normalization factor for microRNA real-time quantitative PCR data and compare its performance to the currently adopted approach. We demonstrate that the mean expression value outperforms the current normalization strategy in terms of better reduction of technical variation and more accurate appreciation of biological changes.

## Abstract 2

The mean expression value: a new method for accurate and reliable normalization of microRNA expression data from RT-qPCR experiments.

## Background

MicroRNAs (miRNAs) are an important class of gene regulators, acting on several aspects of cellular function such as differentiation, cell cycle control and stemness. Not surprisingly, deregulated miRNA expression has been implicated in a wide variety of diseases, including cancer [1]. Moreover, miRNA expression profiling of different tumor entities resulted in the identification of miRNA signatures correlating with patient diagnosis, prognosis and response to treatment [2]. Despite the small size of miRNA molecules, several technologies have been developed that enable high-throughput and sensitive miRNA profiling, such as microarrays [3-8], real-time quantitative PCR (RT-qPCR) [9,10] and bead-based flow cytometry [2]. In terms of accuracy and specificity, RT-qPCR has become the method of choice for measuring gene expression levels, both for coding and non-coding RNAs. However, the accuracy of the results is largely dependent on proper data normalization. As numerous variables inherent to an RT-qPCR experiment need to be controlled for in order to differentiate experimentally induced variation from true biological changes, the use of multiple reference genes is generally accepted as the gold standard for RT-qPCR data normalization [11]. Typically, a set of candidate reference genes is evaluated in a pilot experiment with representative samples from the experimental condition(s). Ideally these candidate reference genes belong to different functional classes, significantly reducing the possibility of confounding co-regulation. In case of miRNA profiling, only few candidate reference miRNAs have been reported [12]. Generally, other small non-coding RNAs are used for normalization. These include both small nuclear RNAs (for example, U6) and small nucleolar RNAs (for example, U24, U26).

Strategies for normalization of high-dimensional expression profiling experiments (using, for example, microarray technology, but recently also transcriptome sequencing) generally take advantage of the huge amount of data generated and often use (almost) all available data points. These strategies range from a straightforward approach based on the mean or median expression value to more complex algorithms such as lowess normalization, quantile normalization or rank invariant normalization [13]. In this study we successfully introduce the mean expression value in a given sample to normalize high-throughput miRNA RT-qPCR data and compare its performance to the currently adopted approach based on small nuclear/nucleolar RNAs. In addition, we provide a workflow for proper data normalization of both large scale (whole miRNome) and small scale miRNA profiling experiments.

## Results

### Stability of the mean miRNA expression

To evaluate the suitability of the mean miRNA expression value as a normalization factor, we profiled 448 miRNAs and controls in a subset of 61 neuroblastoma (NB) tumor samples and 384 miRNAs and controls in 49 T-cell acute lymphoblastic leukemia (T-ALL) samples, 18 leukemias with *EVI1 *overexpression, 8 normal human tissues and 11 normal bone marrow samples using a high throughput miRNA profiling platform based on Megaplex stem-loop RT-qPCR technology in combination with a limited cycle pre-amplification [9,10]. For each of the above mentioned sample sets all 18 available small RNA controls were quantified. For each individual sample, the mean expression value was calculated based on those miRNAs that were expressed according to a Cq detection cut-off of 35 PCR cycles [10] (Cq, or quantification cycle, is the standard name for the Ct or Cp value according to Real-time PCR Data Markup Language (RDML) guidelines [14]). Expression stability of the mean expression value, the small RNA controls and a selection of three miRNAs (miR-17-5p, miR-191 and miR-103) previously proposed as universal reference miRNAs was then assessed for each sample set using the geNorm algorithm [11]. To reduce the risk of including genes that are putatively co-regulated, a number of small RNA controls residing within the same gene cluster were discarded, retaining only one representative small RNA control per cluster. This was the case for RNU44, U47 and U75 on 1q25, and RNU58A and RNU58B on 18q21, of which RNU44 and RNU58A were randomly retained for further analysis. Naturally, only those small RNA controls that are expressed in all samples within a sample set were evaluated for their expression stability.

**Figure 1:**
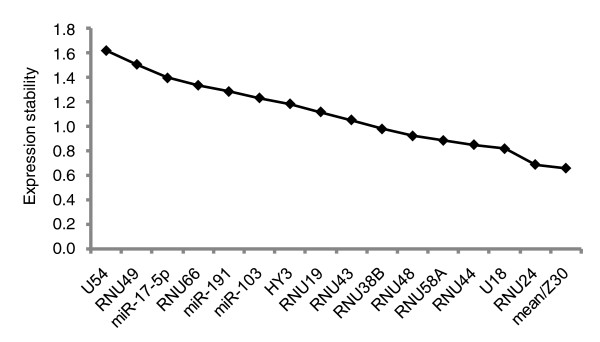
geNorm expression stability plot. Expression stability of 13 different small RNA controls and the mean expression value in the T-cell acute lymphoblastic leukaemia sample set. The mean expression value shows the highest expression stability across all 49 samples analyzed.

geNorm analysis clearly shows that the mean expression value is a suitable normalization factor in the different tissue groups under investigation. In terms of expression stability, the mean expression value is top ranked in the T-ALL samples, the NB samples, the normal human tissues and the normal bone marrow samples when compared to 16, 17, 14 and 18 candidate small RNA controls/miRNAs, respectively (Figure 1 and Additional data file 1). For the leukemia samples with *EVI1 *overexpression the mean expression value ranked second (compared to 17 small RNA controls/miRNAs; Additional data file 1). Several of the high ranking small RNA controls are the same ones proposed by the manufacturer as most suitable for miRNA normalization. The expression stability of one of the so-called universal reference miRNAs (miR-191) proposed by Peltier and Latham [12] equaled that of the mean expression value in the NB sample set. In the other sample sets, none of these three miRNAs performed as well as the mean expression value. When we calculated an alternative mean expression value (only including those miRNAs that are expressed in all samples within a given sample set), it was never as good or better (in terms of suitability as normalization factor) than the mean expression value of all expressed miRNAs. This indicates that the mean expression value more faithfully represents the input amount when all expressed miRNAs per sample are considered. All results obtained with geNorm were independently confirmed with the Normfinder algorithm [15] (data not shown).

### Mean expression value normalization reduces technical variation

**Figure 2:**
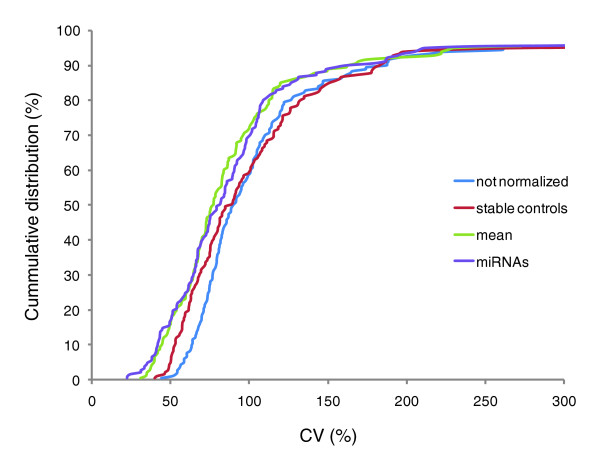
Cumulative distribution of miRNA coefficient of variation (CV) values. The cumulative distribution of miRNA CV values in the neuroblastoma sample set when no normalization is applied (blue), stable RNA control (RNU24, RNU44, RNU58A and RNU6B) normalization is applied (red), mean expression value normalization is applied (green) or normalization with miRNAs/small RNA controls resembling the mean expression value (Z30, RNU24, miR-361, miR-331 and miR-423) is applied (purple).

The variation in gene expression data is a combination of biological and technical variation. The purpose of normalization is to reduce the technical variation within a dataset, enabling a better appreciation of the biological variation. We calculated the coefficient of variation (CV) for each individual miRNA across all samples within a given tissue group and used it as a normalization performance measure. Lower CVs hereby denote better removal of experimentally induced noise [16,17]. Relative expression data were normalized using either the mean expression value of all expressed miRNAs or the mean of the most stable small RNA controls (as identified by geNorm; arithmetic means were calculated in log space). The optimal number of stable controls was determined on the basis of a pairwise variation analysis between subsequent normalization factors using a cut-off value of 0.15 as described in Vandesompele *et al*. [11]. The cumulative distribution of the individual CV values was plotted for both raw (not normalized) and normalized data (Figure 2).

While normalization using stable small RNA controls clearly results in a significant decrease of the CV value in the NB sample set, this shift is only apparent for the 50% least variable miRNAs (paired sample *t*-test, *P *< 0.001; Figure 2 and Additional data file 2). For the 50% most variable miRNAs no significant reduction in variation is observed (*P *= 0.253; Additional data file 2), indicating that elimination of technical variation is restricted to only half of the miRNAs profiled. In contrast, after normalization with the mean expression value there is an overall decrease in variation that is significant both for the 50% least variable (*P *< 0.001) and the 50% most variable (*P *< 0.001) miRNAs (Additional data file 2). Furthermore, a more pronounced reduction in variation is observed compared to stable small RNA control normalization (Figure 2). As true differentially expressed miRNAs predominantly reside in the most variable half of the dataset (50% most variable), only mean expression value normalization is capable of reducing the number of false negatives. Reduction of false positives is possible with both normalization strategies but to different extents as mean expression value normalization results in a stronger decrease of technical variation for the 50% least variable miRNAs. Similar results were obtained for the other sample sets (Additional data file 3 and data not shown).

### Mean expression value normalization identifies true biological changes in patient samples

While the mean expression value is the best ranked normalization factor and significantly reduces technical variation, the question remains how different normalization strategies affect biological changes. To address this issue, we evaluated differential expression of the miRNAs belonging to the mir-17-92 cluster in the NB sample set. The miR-17-92 cluster contains a total of six different miRNAs (miR-17, miR-18a, miR-19a, miR-20a, miR-19b and miR-92) and has recently been shown to be a direct target of the MYC family of transcription factors using chromatin immunoprecipitation (ChIP) [18,19]. In NB cells, MYCN directly binds to the miR-17-92 promoter, resulting in an activation of mir-17-92 expression [18]. Accordingly, NB cells with amplification and activation of the *MYCN *oncogene display elevated mir-17-92 expression [18,20,21].

To confirm MYCN binding to the miR-17-92 promoter, we performed ChIP-chip experiments using a MYCN-specific antibody in three different NB cell lines, Kelly, IMR5 and WAC2. To assess whether transcripts from this region are actively transcribed and elongated, we additionally analyzed histone marks for active transcription (H3K4me3), repression (H3K27me3), and elongation (H3K36me3) together with MYCN binding. In all tested NB cell lines, binding of MYCN was preferentially found to the miR-17-92 promoter region encompassing the two canonical e-boxes upstream of miR-17 (Additional data file 4). Furthermore, MYCN binding to the miR-17-92 promoter was strongly associated with histone marks for active transcription (H3K4me3) and elongation (H3K36me3) (Additional data file 4). To confirm the MYCN ChIP-chip data, we performed ChIP-qPCR on ChIP samples from WAC2 and IMR5 cells. Both promoter fragments were enriched in the two cell lines under investigation, with fold changes comparable to that of the MDM2 positive control, confirming direct MYCN binding to the miR-17-92 promoter (Additional data file 5).

**Figure 3:**
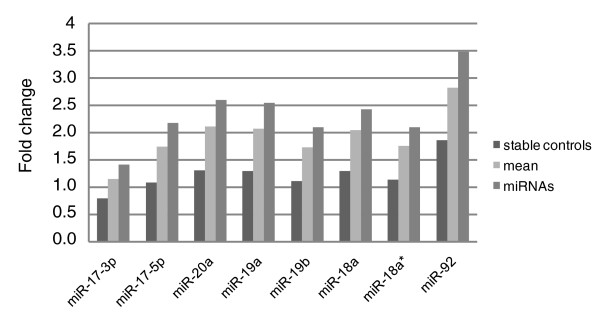
Differential miR-17-92 expression in neuroblastoma tumor samples. Average fold change expression difference of eight different miRNAs residing within the miR-17-92 cluster in MYCN amplified neuroblastoma samples compared to MYCN single copy neuroblastoma samples. Fold changes were calculated upon stable small RNA control (RNU24, RNU44, RNU58A and RNU6B) normalization (dark grey), mean expression value normalization (light grey) and normalization with miRNAs that resemble the mean expression value (miR-425, miR-191 and miR-125a; medium grey.

To assess the impact of different normalization strategies on differential miR-17-92 expression, the NB sample set, consisting of 22 *MYCN *amplified (MNA) and 39 *MYCN *single copy (MNSC) tumor samples, was normalized using either the mean expression value or the stable small RNA controls. Differential miR-17-92 expression was then evaluated by means of the average fold change in expression between the MNA and MNSC tumor samples (Figure 3). When the data are normalized using the stable small RNA controls, none of the 8 miRNA transcripts that were analyzed reach a 2-fold expression difference and only one miRNA, miR-92, exceeds a 1.5-fold expression difference (fold change = 1.85). Moreover, miR-92 is the only miRNA from the miR-17-92 cluster with a significant differential expression between MYCN amplified and MYCN single copy tumor samples (Mann-Whitney, Benjamini-Hochberg multiple testing correction, *P *< 0.05).

These results are not in line with previous studies reporting differential expression of multiple miRNAs from the miR-17-92 cluster nor do they match our findings, and those of others, regarding the direct interaction between MYCN and the miR-17-92 promoter [18]. Furthermore, our analysis of histone markers bound to the region is more in line with an actively transcribed entire miR-17-92 cluster in MYCN amplified cell lines. When the same dataset is normalized with the mean expression value, 7 miRNAs reach a 1.5-fold expression difference and half of the miRNAs exceed the 2-fold expression difference. All but one miRNA, mir-17-3p, were found to be significantly differentially expressed between MNA and MNSC tumors (Mann-Whitney, Benjamini-Hochberg multiple testing correction, *P *< 0.05). A recent study by Chen and Stallings [20] reports on differential miRNA expression between MNA and MNSC tumors, measured by stem-loop RT-qPCR. Here, only one miRNA from the five miR-17-92 miRNAs that were evaluated was reported as significantly upregulated in the MNA tumor samples. In that study, miRNA expression data were normalized using two small RNA controls, RNU19 and RNU66. We reanalyzed the same dataset and applied the mean expression value normalization strategy. As expected, all but one miRNA, miR-17-3p, were significantly upregulated in the MNA tumors (Mann-Whitney, Benjamini-Hochberg multiple testing correction, *P *< 0.05; data not shown).

To ascertain that these observations are not restricted to miR-17-92, we identified an additional MYCN regulated miRNA cluster using ChIP-chip. MiR-181a-1 and miR-181b-1 are located within 500 bp of each other and show strong MYCN binding in two MNA NB cell lines, Kelly and IMR5. MYCN binding was strongly associated with histone marks for transcription (H3K4me3) and elongation (H3K36me3) (Additional data file 6). Accordingly, mir-181a and mir-181b expression should be upregulated in MNA NB tumor samples. Upon mean expression value normalization, both miRNAs exceed the 1.5-fold expression difference (FC_mir-181a _= 2.28, FC_mir-181b _= 1.67). Upon normalization with stable small RNA controls, only miR-181a has a fold change above 1.5-fold (FC_mir-181a _= 1.59). For miR-181b, no change in expression could be detected (FC_mir-181b _= 1.14). These results confirm that the ability of mean expression normalization to extract true biological variation from a dataset is not limited to miR-17-92.

### Mean expression value normalization identifies true biological changes in cell lines

While small RNA control normalization fails to identify differential miR-17-92 expression in patient tumor samples, it has been successfully applied by Fontana and colleagues [18] to detect differential miR-17-92 expression in NB cell lines. To evaluate our method in cell lines, we measured miRNA expression in two NB cell lines also used by Fontana and colleagues, one MYCN single copy (SK-N-AS) and one MYCN amplified (IMR-32). MiR-17-92 fold induction upon mean expression value normalization was consistently higher compared to fold inductions reported by Fontana and colleagues. Further, fold changes for all 5 miRNAs exceed the 1.5-fold expression difference whereas with small RNA control normalization this is only true for 4 out of 5 miRNAs (Additional data file 7).

### Mean expression value normalization reduces false positive MYCN downregulated miRNAs

We sought further support for our new normalization strategy by investigating the overall differential miRNA expression in the two subsets of NB tumor samples. miRNAs that were not expressed in all samples were excluded from the analysis to avoid over- or underestimation of fold changes. Upon normalization with stable small RNA controls, we found an average miRNA expression fold change of 0.756, suggesting that the majority of the miRNAs were downregulated in the MNA tumor samples. Indeed, 89.1% of the miRNAs displaying a minimum 1.5-fold expression difference are expressed at lower levels in the MNA tumor samples (Additional data file 8) indicating a bias towards the identification of downregulated miRNAs. When normalizing with the mean expression value the average miRNA expression fold change levels out to a value of 1.036, representing a more balanced situation. Here, only 57.6% of the differentially expressed miRNAs are downregulated in the MNA tumor samples. Moreover, the fold change expression difference for the 10% most downregulated miRNAs, identified after stable small RNA control normalization, remains largely unaffected upon normalization with the mean expression value (Additional data file 9), suggesting that this normalization strategy more adequately reduces the number of false positive MYCN downregulated miRNAs compared to stable small RNA control normalization. This is in perfect agreement with the larger reduction of variation obtained with mean expression value normalization (see above).

### miRNAs resembling the mean

The use of the mean expression value for data normalization implies that a large number of genes are profiled (450 or 384 in this study). Such screening experiments are often performed in an initial phase but almost never in subsequent validation studies that focus on a limited number of miRNAs. We therefore assessed whether we could identify miRNAs or small RNA controls that resemble the mean expression value and whether their geometric mean could be successfully used to mimic mean expression value normalization. After log transformation, we calculated the geNorm pairwise variation V value to determine robust similarity in expression of a given gene with the mean expression value. For each tissue group the optimal number of miRNAs/small RNA controls was selected and the geometric mean of their relative expression values was used for normalization (Table 1). In the NB sample set, the reduction in technical variation is highly similar to that obtained after mean expression value normalization, as illustrated by the cumulative distribution plot of miRNA CV values (Figure 2). Here also, the overall decrease in variation is significant both for the 50% least variable (*P *< 0.001) and the 50% most variable (*P *< 0.001) miRNAs (Additional data file 2). Similar results were obtained for other sample sets (Additional data file 3). These findings indicate that the geometric mean of a limited number of carefully selected miRNAs/small RNA controls that resemble the mean can be successfully used for normalization of gene expression profiling experiments in follow-up studies where only a limited number of miRNA molecules are studied.

**Table 1:** Selection of miRNAs that resemble the mean expression value

Neuroblastoma	T-ALL	EVI1 leukemia	Normal tissue	Normal bone marrow
miR-425*	Z30^†^	miR-191*	miR-572*	miR-140*
miR-191*	RNU24^†^	miR-140*	let-7f*	miR-30c*
miR-125a*	miR-361*	miR-16*	miR-632*	miR-328*
	miR-331*		miR-339*	
	miR-423*		RPL21^†^	

*Human mature miRNA. ^†^Small RNA control. T-ALL, T-cell acute lymphoblastic leukaemia.

**Figure 4:**
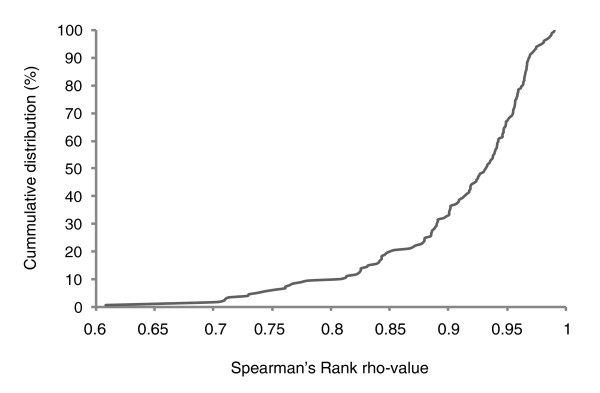
Cumulative distribution of Spearman's Rank rho values. The cumulative distribution of the Spearman's Rank rho values for each individual miRNA in the neuroblastoma sample set. The rho-values represent the degree of correlation between the miRNA expression profile upon mean expression value normalization or normalization with miRNAs resembling the mean expression value.

We further investigated the use of these stable miRNAs/small RNA controls for normalization by evaluating the impact on differential miRNA expression. In the NB sample set, differential expression of the miR-17-92 cluster is significant for all but one miRNA, with fold changes highly similar to those obtained upon normalization with the mean expression value (Figure 3). Moreover, miRNA expression profiles generated with both normalization strategies are significantly correlated as over 90% of all miRNAs display a correlation coefficient above 0.8 and 65% have a correlation coefficient above 0.9 (Spearman's Rank rho value; Figure 4). Similar results were obtained with other sample sets (data not shown).

### Normalization using miRNAs that resemble the mean is platform independent

Finally, the correlation between both normalization strategies was validated on an independent dataset of microarray miRNA expression data from 12 NB cell lines. Probe intensities were log transformed and the mean expression value was calculated for each array. Subsequently, miRNAs with expression levels correlating to the mean expression value were identified as outlined above and the best miRNAs were selected for further normalization. Log intensities were normalized using either the mean expression value of all probes or the mean expression of the selected miRNAs. Hierarchical clustering of a compiled dataset consisting of mean and miRNA normalized samples reveals a high correlation between each sample pair as pairs consistently cluster together (Additional data file 10). Over 95% of all miRNAs show a correlation coefficient above 0.7 and 87% have a correlation coefficient above 0.8 (Spearman's Rank rho value). These results illustrate that normalization using miRNAs that resemble the mean expression value is platform independent and closely mimics normalization using the mean expression value.

## Discussion

In this study we present the use of the mean miRNA expression value as a new method for miRNA RT-qPCR data normalization. This method was validated across different independent datasets and clearly outperforms the current normalization strategy that is based on the use of endogenous small RNA controls. Our results demonstrate that the mean expression value of all expressed miRNAs is characterized by high expression stability, according to geNorm analysis, resulting in an adequate removal of technical variability, as measured by the CV of normalized expression values. While mean normalization results in reduction of noise over all expressed miRNA, stable small RNA control normalization only achieves this for the 50% least variable miRNAs. Interestingly, the mean expression value of all expressed miRNAs performs better than one based on only those miRNAs that are expressed in all samples. This suggests a more accurate representation of input RNA fluctuations when all miRNAs are considered. Furthermore, the mean expression value is more stable than a set of three miRNAs (miR-103, miR-191 and miR-17-5p) previously proposed as universal reference miRNAs [12]. Only in the NB sample set could we confirm stable expression of miR-191 and miR-103. miR-17-5p is activated by MYC transcription factors, which results in mir-17-5p overexpression in tumors with activated MYC signaling [18,19]. Moreover, mir-17-5p is located on 13q31.3, a region frequently amplified in B-cell lymphomas, resulting in elevated mir-17-5p expression [22]. Accordingly, mir-17-5p does not qualify as a proper candidate reference miRNA.

Several studies report on the use of synthetic RNA or miRNA molecules as spike-in controls for mRNA/miRNA expression data normalization [23-26]. While these kind of controls have value in assay validation and quality control, they only correct for extraction efficiency (when added to the cells prior to RNA isolation) or reverse transcription efficiency (when added to the RNA) differences when using them for normalization. As such, they do not control for all experimental variability, and are not assumption-free as it is assumed that the experimenter starts with the same quantity of equal quality template. Normalization factors that are based on endogenous small RNA molecules, such as the small RNA controls, miRNA molecules, or the mean miRNA expression value, are therefore preferred.

To assess the impact of small RNA control, miRNA or mean expression value normalization on biological variation, we studied the differential expression of the miR-17-92 cluster in the NB dataset, consisting of samples with and without *MYCN *amplification. Because differential expression of this miRNA cluster has been repeatedly documented, both in the context of MYC family transcription factors and in the context of NB tumors [18,19], we reasoned that it could serve as an excellent positive control. Strikingly, only 1 of the 8 miR-17-92 miRNAs analyzed showed an expression fold change of at least 1.5-fold after small RNA control normalization. A 1.5-fold expression difference cut-off is based on several miRNA profiling studies confirming that subtle changes in miRNA expression, such as a 1.5-fold difference, can have a significant impact on the biology of the cell [27-32]. As a consequence, a proper normalization strategy that enables detection of these small changes is of the utmost importance. Upon mean expression value normalization, seven miRNAs exceeded the 1.5-fold expression difference. For one miRNA, mir-17-3p, no expression difference was detected; however, the status of mir-17-3p as a functional miRNA is still controversial [19,33,34].

We and others have shown that MYC transcription factors actively bind to the miR-17-92 promoter [18,19]. In addition, we here describe histone marks associated with active transcription and elongation that are not restricted to a single miRNA but encompass the entire set of miRNAs from the miR-17-92 cluster. Taken together with the fact that the miR-17-92 cluster is transcribed as a single transcript (pri-miR-17-92) [22], most likely all miRNAs should be activated in the MNA NB cells. The results obtained with mean expression value normalization are best in line with this hypothesis. While small RNA control normalization in the clinical tumor samples appears not to be affective, in cultured cells this strategy is capable of detecting differential expression for the majority of the mir-17-92 miRNAs [18]. This could be explained by the degree of heterogeneity of the sample set under consideration. Tumor samples are typically more heterogeneous than cultured cells and, therefore, require a more robust normalization strategy that is able to reduce this variability.

Apart from differential miR-17-92 expression, we also evaluated global miRNA expression in the NB tumors with regard to *MYCN *amplification status. Upon normalization with stable small RNA controls, differential miRNA expression was highly unbalanced, with 89.1% of all differentially expressed miRNAs being downregulated. In contrast, literature reports on differential mRNA expression with regard to *MYCN *amplification status suggest a more balanced situation. From a total of 678 coding genes that have been described as differentially expressed, 63% are upregulated and 37% are downregulated [35]. The unbalanced differential miRNA expression that is observed upon stable small RNA control normalization is most likely caused by an unbalanced normalization factor that hypercorrects miRNA expression in MYCN amplified tumors. Indeed, we calculated a significantly higher normalization factor for amplified versus not-amplified tumors (data not shown). Furthermore, small RNA controls and miRNAs are transcribed by different RNA polymerases [36], possibly making these small RNA controls improper normalizers for miRNA expression. This has been well established for mRNA expression normalization as ribosomal RNAs, which are transcribed by RNA polymerase I, are often poor and unstable normalizers for mRNAs [11], which are transcribed by RNA polymerase II. Mean expression value normalization is based on the expression of miRNAs and results in a more balanced differential miRNA expression with only 57.6% downregulated miRNAs.

Importantly, mean expression value normalization is only valid if a large number of miRNAs are profiled. However, for small scale experiments, typically focusing on a selection of miRNAs, this is not the case. To overcome this problem, we have shown that it is possible to identify miRNAs and small RNA controls that resemble the mean expression value. Our results indicate that a normalization factor based on the selection of miRNAs/small RNA controls resembling the mean expression value performs equally well as the mean expression value itself. We therefore propose a workflow consisting of a pilot experiment in which miRNAs/small RNA controls can be identified that resemble the mean expression value. Subsequently, these can be used for proper normalization of miRNA expression in targeted small scale experiments, focusing on only a limited number of genes. miRNA gene expression studies in which no prior whole miRNome expression profiling can be performed should be preceded by a careful selection of the most stable small RNA controls. In this case, cautious interpretation of the data is warranted.

## Conclusions

A proper normalization strategy is a crucial aspect of the RT-qPCR data analysis workflow. For large scale miRNA expression profiling studies we have shown that mean expression value normalization outperforms the current normalization strategy that makes use of small RNA controls. For those experiments focusing on a limited number of miRNAs we propose a workflow that is based on the selection of miRNAs/small RNA controls that resemble the mean expression value. This strategy is innovative, straightforward and universally applicable and enables a more accurate assessment of relevant biological variation from a miRNA RT-qPCR experiment.

## Materials and methods

### Samples

A total of 147 samples from 5 different tissue groups were used in this study, including 61 NB tumors, 49 T-ALL samples, 18 leukemias with EVI1 overexpression, 8 normal human tissue samples (brain, colon, heart, kidney, liver, lung, breast, adrenal gland) and 11 normal bone marrow samples. RNA samples from the normal human tissue group were obtained from Stratagene (Cedar Creek, TX, USA). NB tumor RNA was isolated using the miRNeasy mini kit (Qiagen, Valencia, CA, USA) according to the manufacturer's instructions. RNA from leukemic and normal bone marrow samples was isolated as described previously [37]. For each sample, total RNA integrity was measured using the Experion (Bio-Rad, Hercules, CA, USA) and evaluated through the RNA quality index; for all samples this was higher than 5.

### RDML data and MIQE guidelines

Compliance of qPCR experiments with the MIQE (Minimum Information for Publication of Quantitative Real-Time PCR Experiments) guidelines [38,39] is listed in the MIQE checklist (Additional data file 11). Raw miRNA expression, experimental annotation and sample annotation are available in the RDML data format [14,40] (Additional data file 12).

### Cell culture

Twelve NB cell lines (NGP, IMR-32, SMS-KAN, SK-N-BE(2c), LAN-5, LAN-6, SK-MYC2, SK-N-AS, SK-N-SH, NBL-S, SK-N-FI and CLB-GA) were cultured in RPMI 1640 medium (Invitrogen, Carlsbad, CA, USA) supplied with 15% fetal calf serum, 1% penicillin/streptomycin, 1% kanamycin, 1% glutamine, 2% HEPES (1 M), 1% sodiumpyruvate (100 nM) and 0.1% beta-mercapto (50 nM). At 80% confluence, cells were harvested by scraping for total RNA isolation (miRNeasy, Qiagen).

### MicroRNA profiling

miRNA expression was measured as described previously [10]. Briefly, 20 ng of total RNA was reverse transcribed using the Megaplex RT stem-loop primer pool (Applied Biosystems, Foster City, CA, USA), enabling miRNA specific cDNA synthesis for 430 different human miRNAs and 18 small RNA controls. Subsequently, Megaplex RT product was pre-amplified by means of a 14-cycle PCR reaction with a miRNA specific forward primer and universal reverse primer to increase detection sensitivity. Finally, a 1,600-fold dilution of pre-amplified miRNA cDNA was used as input for a 40-cycle qPCR reaction with miRNA specific hydrolysis probes and primers (Applied Biosystems). All reactions were performed on the 7900 HT (Applied Biosystems) using the gene maximization strategy [41]. Raw Cq values were calculated using the SDS software version 2.1 applying automatic baseline settings and a threshold of 0.05. For further data analysis, only those miRNAs with a Cq value equal to or below 35 (representing single molecule template detection [10]) were taken into account. For NB tumor samples all 448 miRNAs and small RNA controls were profiled. RT-qPCR assays were spread across two 384-well plates. Inter-run variation was accounted for by equalizing the mean Cq-value of the 18 small RNA controls that were profiled in both plates. For the remaining samples 366 miRNAs and 18 small RNA controls were profiled in a single 384-well plate.

### Selection of stable normalizers

Assessing gene expression stability of putative normalizer genes was done using two different algorithms, geNorm [11] and Normfinder [15]. Raw Cq values were transformed to linear scale before analysis. Normalization factors were calculated as the geometric mean of the expression of the stable normalizers [41]. Selection of the optimal number of stable normalizers was based on geNorm's pairwise variation analysis between subsequent normalization factors using a cut-off value of 0.15 for the inclusion of additional normalizers [11].

### Selection of miRNAs/small RNA controls that resemble the mean expression value

For robust and unbiased selection of genes whose expression level best correlates with the mean expression level, we used the geNorm V value [11]. In brief, for each miRNA and small RNA control we calculated the difference between its Cq value and the average Cq value of all expressed genes, per sample, within a given sample set. Next, the standard deviation of these differences was determined for every miRNA and small RNA control. The miRNAs or small RNA controls with the lowest standard deviation most closely resemble the mean expression value. The optimal number of miRNAs/small RNA controls for normalization was determined upon geNorm analysis of the ten best ranked normalizers. To avoid including miRNAs that are putatively co-regulated, we determined their genomic location and excluded those miRNAs that are located within 2 kb of each other using miRGen [42]. Co-regulated miRNAs were replaced by the next best ranked miRNA.

### Chromatin immunoprecipitation

Immunoprecipitation was performed as described previously using 10 μg of MYCN (Santa Cruz, sc-53993, Santa Cruz, CA, USA) antibodies [43]. Histone marks for active transcription (H3K4me3; Abcam, ab8580, Cambridge, MA, USA), repression (H3K27me3; Upstate, 07-449, Lake Placid, NY, USA), and elongation (H3K36me3; Abcam, ab9050) were assessed together with MYCN binding. ChIP-DNA templates from Kelly, IMR5, WAC2 cells using MYCN, H3K4me3, H3K27me3 and H3K36me3 were amplified for DNA microarray analysis (Agilent Human Promoter ChIP-chip Set 244 K, Santa Clara, CA, USA) using the WGA (whole genome amplification) (Sigma, St. Louis, MO, USA) method as previously described [43]. DNA labeling, array hybridization and measurement were performed according to Agilent mammalian ChIP-chip protocols. For the visualization of ChIP-chip results, the cureos package version 0.2 for R was used (available upon request).

Real-time ChIP-qPCR was performed using SYBR Green I detection chemistry (Eurogentec, Seraing, Belgium) on a LightCycler480 (Roche, Basel, Switzerland). Primer sequences for MYCN binding sites in the mir-17-92 and MDM2 promoter regions were described previously [19,44]. Signals were normalized based on the average abundance of three non-specific genomic regions in the ChIP samples using qBase*Plus *version 1.1 software [45]. Fold enrichment in the MYCN precipitated samples was calculated relative to the input sample and compared to that of a fourth non-specific region. All primer sequences are available in the public RTprimerDB database [46] (gene (RTPrimerDB-ID): miR-17-92 promoter A (7796), miR-17-92 promoter B (7797), MDM2 promoter (7798), non-specific region 1 (7799), non-specific region 2 (7800), non-specific region 3 (7801), non-specific region 4 (7802)) [47].

### Locked nucleic acid microarrays

In total, 5 μg of total RNA was hybridized to immobilized locked nucleic acid-modified capture probes according to Castoldi *et al*. [48]. Background- and flag-corrected median intensities were log transformed and normalized according to the mean signal of each array.

### Hierarchical clustering

Hierarchical clustering of the miRNA expression data was performed using Spearman's rank correlation as the sample and gene distance measure and pairwise complete linkage as implemented in the Genepattern 2.0 software [49].

## Abbreviations

ChIP: chromatin immunoprecipitation; CV: coefficient of variation; miRNA: microRNA; MNA: MYCN amplified; MNSC: MYCN single copy; NB: neuroblastoma; RDML: Real-time PCR Data Markup Language; RT-qPCR: real-time quantitative PCR; T-ALL: T-cell acute lymphoblastic leukaemia.

## Authors' contributions

PM carried out the miRNA profiling experiments and data analysis and drafted the manuscript. PVV and ADW performed miRNA profiling experiments. DM and FW are responsible for MYCN ChIP-on-chip data. FS and JV conceived the study and participated in its design and coordination. All authors read and approved the final manuscript.

## Additional data files

The following additional data are available with the online version of this paper: a figure showing geNorm expression stability plots (Additional data file 1); a figure showing the mean miRNA CV value in the neuroblastoma sample set (Additional data file 2); a figure showing the cumulative distribution of miRNA CV values (Additional data file 3); a figure showing ChIP-chip results for the miR-17-92 cluster (Additional data file 4); a figure showing ChIP-qPCR results for the miR-17-92 cluster (Additional data file 5); a figure showing ChIP-chip results for the miR-181a-1/miR-181b-1 cluster (Additional data file 6); a figure showing miR-17-92 expression in neuroblastoma cell lines (Additional data file 7); a figure showing overall differential miRNA expression in the neuroblastoma sample set (Additional data file 8); a figure showing fold change expression difference correlation for MYCN downregulated miRNAs (Additional data file 9); a figure showing hierarchical clustering of neuroblastoma cell lines based on miRNA expression (Additional data file 10); a table listing the MIQE checklist (Additional data file 11); a collection of RDML files containing miRNA expression for all data sets (Additional data file 12).

## Supplementary Material

Additional data file 1 Expression stability of small RNA controls and the mean expression value in **(a) **the neuroblastoma sample set, **(b) **the leukemias with EVI1 overexpression, **(c) **the normal bone marrow samples and **(d) **the normal human tissues. Click here for file

Additional data file 2 Mean miRNA CV value for **(a) **the 50% least variable and **(b) **50% most variable miRNAs when no normalization is applied, stable small RNA control normalization is applied, mean expression value normalization is applied or normalization with miRNAs/small RNA controls resembling the mean is applied. **(a) **All three normalization strategies result in a significant decrease of the mean CV value. **(b) **Only mean expression value normalization and normalization with miRNAs/small RNA controls resembling the mean result in a significant decrease of the mean CV value. Stable small RNA controls for the T-ALL samples: RNU24, RNU44, RNU48, RNU58A, U18 and Z30; for the leukemias with EVI1 overexpression: RNU6B, RNU24 and RNU58A; for the normal bone marrow samples: RNU44, RNU24 and RNU48; and for the normal human tissues: RPL21, RNU38B and RNU24. MiRNAs/small RNA controls that resemble the mean expression value are listed in Table 1. Click here for file

Additional data file 3 The cumulative distribution of miRNA CV-values in **(a) **the T-ALL sample set, **(b) **the leukemias with EVI1 overexpression, **(c) **the normal bone marrow samples and **(d) **the normal human tissues when no normalization is applied (blue), stable RNA control normalization is applied (red), mean expression value normalization is applied (green) or normalization with miRNAs resembling the mean expression value is applied (purple). Stable small RNA controls for the T-ALL samples: RNU24, RNU44, RNU48, RNU58A, U18 and Z30; for the leukemias with EVI1 overexpression: RNU6B, RNU24 and RNU58A; for the normal bone marrow samples: RNU44, RNU24 and RNU48; for the normal human tissues: RPL21, RNU38B and RNU24. MiRNAs/small RNA controls that resemble the mean expression value are listed in Table 1. Click here for file

Additional data file 4 ChIP-chip results of the miR-17-92 cluster are given for Kelly, IMR5, and WAC2. Oligonucleotide position is given as bars according to the chromosomal localization. Color coding of the bars represents the log2 ratios MYCN versus input from ChIP-chip experiments, were red means positive and green negative values. Histone marks for active transcription (H3K4me3), repression (H3K27me3) and enlongation (H3K36me3) as measured by ChIP-chip are given together with MYCN binding using the same color coding. miRNA transcript information (miRBase version 11.0), CpG islands, and conservation among 28 species were implemented for the region as given by the respective annotation tracks deposited in the UCSC database (Hg 18, release March 2006). Position of canonical (CACGTG) and non-canonical E-boxes from *in silico *scanning of the respective sequence is given. Grey coding for results of the positional weight matrix (PWM) scan represents the *P*-values of the 12 bp MYCN binding motif from the TRANSFAC database. Red line = median log2 ratio MYCN versus input as calculated for each chromosome individually. Click here for file

Additional data file 5 Fold enrichment of specific and non-specific genomic regions in the MYCN precipitated samples compared to the input sample as determined by qPCR. MiR-17-92 promoter A and miR-17-92 promoter B are two MYCN specific e-box containing regions in the miR-17-92 promoter. MDM2 promoter is a MYCN specific e-box containing region in the MDM2 promoter and serves as a positive control. The negative control is a non-specific, non e-box containing genomic region. Click here for file

Additional data file 6 ChIP-chip results of the miR-181a-1/miR-181b-1 cluster are given for Kelly, IMR5, and WAC2. Oligonucleotide position is given as bars according to the chromosomal localization. Color coding of the bars represents the log2 ratios MYCN versus input from ChIP-chip experiments, were red means positive and green negative values. Histone marks for active transcription (H3K4me3), repression (H3K27me3) and enlongation (H3K36me3) as measured by ChIP-chip are given together with MYCN binding using the same color coding. miRNA transcript information (miRBase version 11.0), CpG islands, and conservation among 28 species were implemented for the region as given by the respective annotation tracks deposited in the UCSC database (Hg 18, release March 2006). Position of canonical (CACGTG) and non-canonical E-boxes from *in silico *scanning of the respective sequence is given. Grey coding for results of the positional weight matrix (PWM) scan represents the *P*-values of the 12 bp MYCN binding motif from the TRANSFAC database. Red line = median log2 ratio MYCN versus input as calculated for each chromosome individually. Click here for file

Additional data file 7 Relative expression of miR-17-5p, miR-18a, miR-19a, miR-20a and miR-92a in one MYCN single copy cell line (SK-N-AS) and one MYCN amplified cell line (IMR-32) upon mean expression value normalization. Relative expression values were rescaled to those in SK-N-AS. Click here for file

Additional data file 8 Average fold change expression difference of all miRNAs with an expression below the Cq cutoff of 35 PCR cycles in MYCN amplified neuroblastoma samples compared to MYCN single copy neuroblastoma samples. Fold changes were calculated upon stable small RNA control normalization (black) and mean expression value normalization (orange). Plotted fold changes are log_2_-based and sorted from positive (upregulated in MYCN amplified tumor samples) to negative (downregulated in MYCN amplified tumor samples). Dashed lines represent a two-fold expression difference. Arrows indicate the threshold between up- and downregulated miRNAs for both normalization strategies (the number of up- and downregulated miRNAs is indicated left and right of each arrow, respectively). Click here for file

Additional data file 9 Correlation plot showing the average fold change expression difference for the 10% most downregulated miRNAs in MYCN amplified tumors compared to MYCN single copy tumors upon stable small RNA control normalization (x-axis) and mean expression value normalization (y-axis). Both axes are log_2_-based. The corresponding trend line has a coefficient of determination of 0.973 (R^2^), a slope approaching 1 and a Y-intercept of 0.449. Click here for file

Additional data file 10 Heatmap representing a hierarchical clustering analysis of 24 paired samples based on their miRNA expression profiles. Each sample pair consists of a different neuroblastoma cell line for which the miRNA expression was normalized with the mean expression value or with miRNAs resembling the mean expression value. Cell lines are numbered from 1 to 12. The tag represents the applied normalization strategy (M stands for mean expression value normalization, m for normalization with miRNAs resembling the mean expression value). Click here for file

Additional data file 11 Compliance of qPCR experiments with the MIQE guidelines. Click here for file

Additional data file 12 RDML files containing miRNA expression and a sample annotation for each sample set. Click here for file
