# Supplementary figures and images for: A novel and universal method for microRNA RT-qPCR data normalization
Source: Genome Biol. 2009 Jun 16;10(6):R64. doi: 10.1186/gb-2009-10-6-r64 (PMC2718498; doi:10.1186/gb-2009-10-6-r64)

A

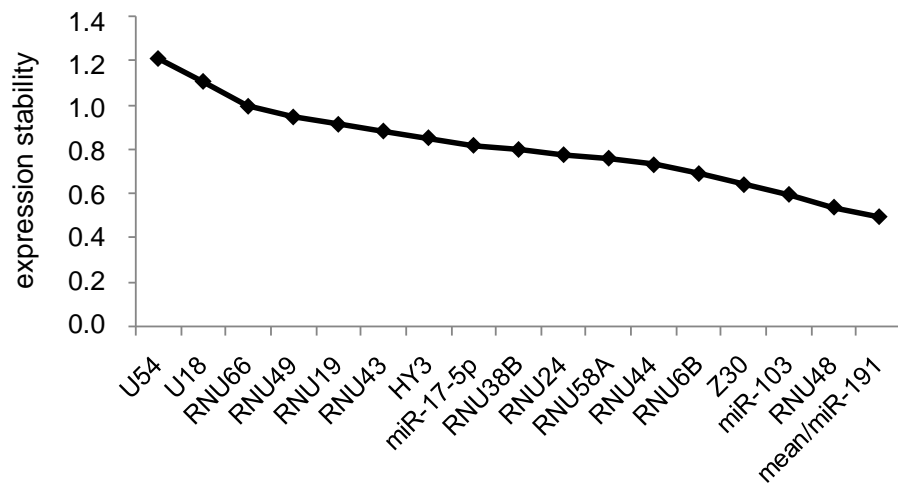

B

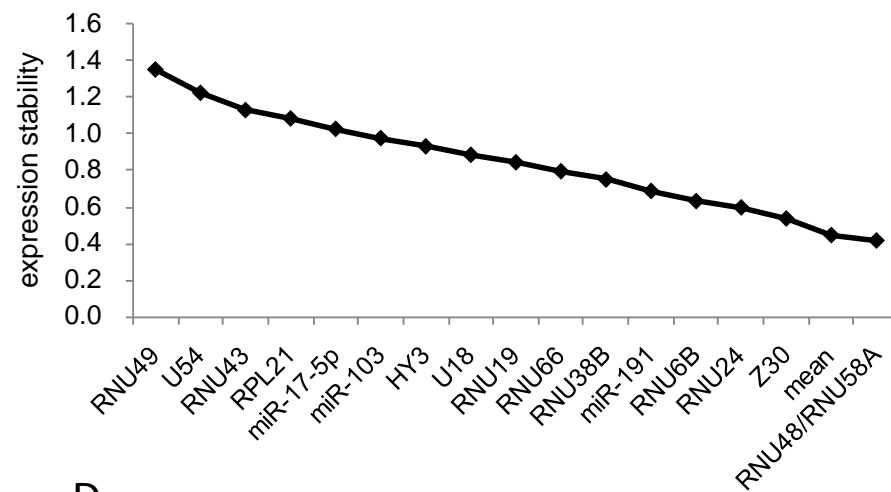

C

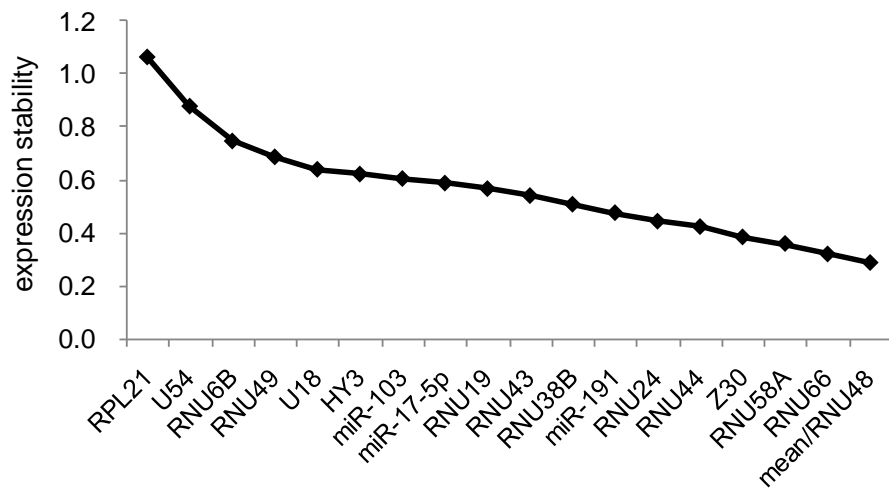

D

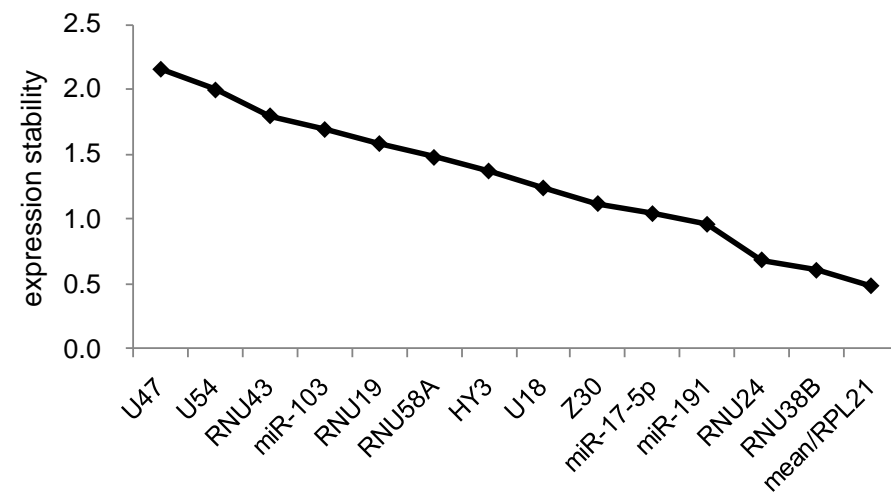

Supplement: Additional data file 1 — Expression stability of small RNA controls and the mean expression value in (a) the neuroblastoma sample set, (b) the leukemias with EVI1 overexpression, (c) the normal bone marrow samples and (d) the normal human tissues. [file gb-2009-10-6-r64-S1.pdf]

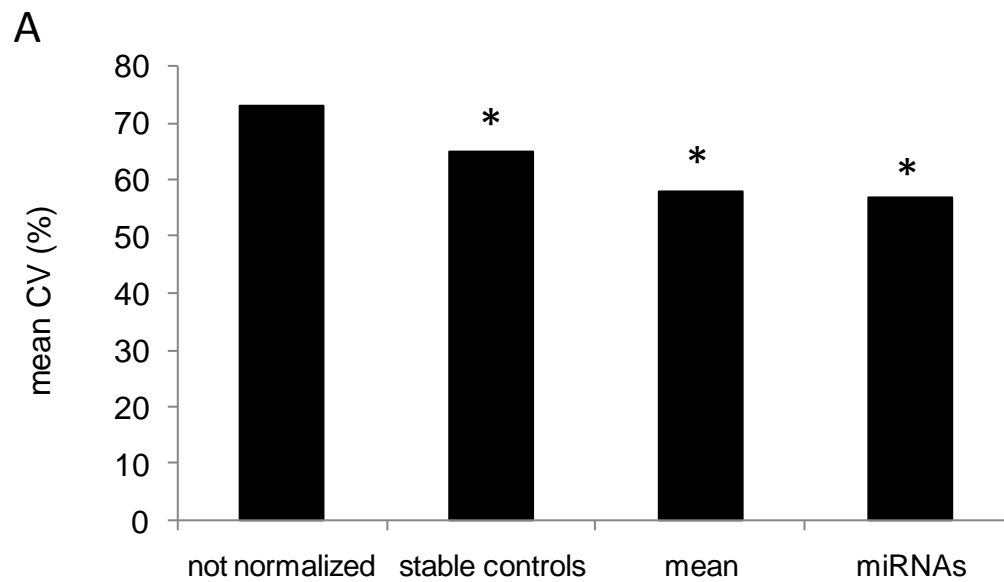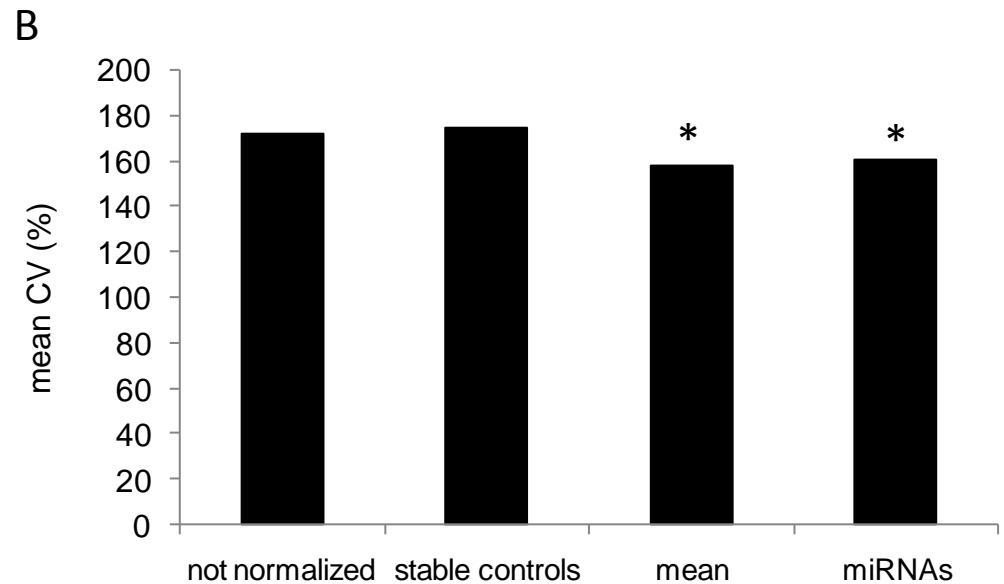

Supplement: Additional data file 2 — Mean miRNA CV value for (a) the 50% least variable and (b) 50% most variable miRNAs when no normalization is applied, stable small RNA control normalization is applied, mean expression value normalization is applied or normalization with miRNAs/small RNA controls resembling the mean is applied. (a) All three normalization strategies result in a significant decrease of the mean CV value. (b) Only mean expression value normalization and normalization with miRNAs/small RNA controls resembling the mean result in a significant decrease of the mean CV value. Stable small RNA controls for the T-ALL samples: RNU24, RNU44, RNU48, RNU58A, U18 and Z30; for the leukemias with EVI1 overexpression: RNU6B, RNU24 and RNU58A; for the normal bone marrow samples: RNU44, RNU24 and RNU48; and for the normal human tissues: RPL21, RNU38B and RNU24. MiRNAs/small RNA controls that resemble the mean expression value are listed in Table 1. [file gb-2009-10-6-r64-S2.pdf]

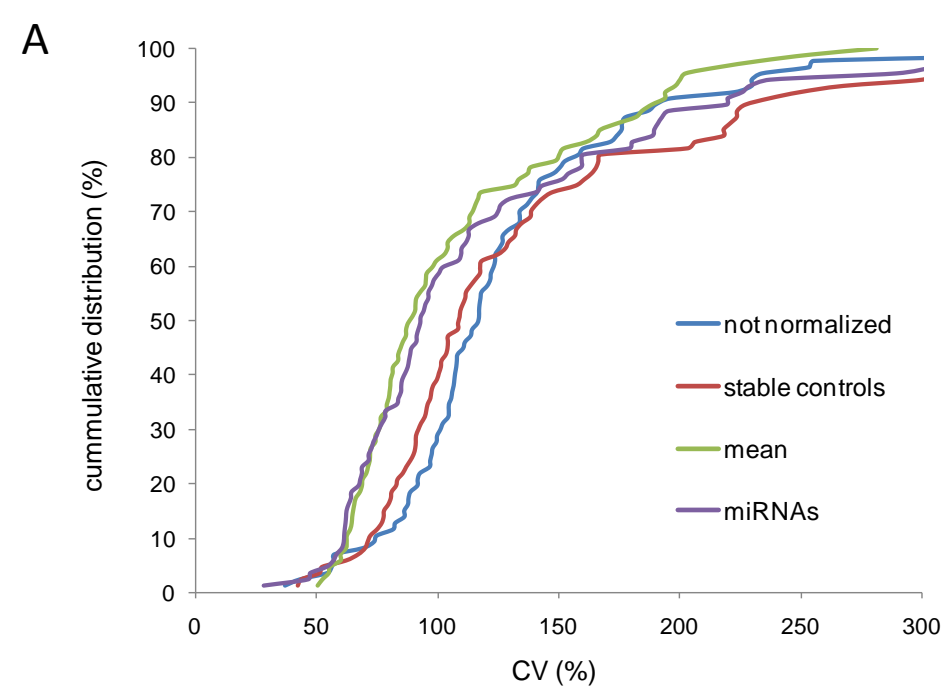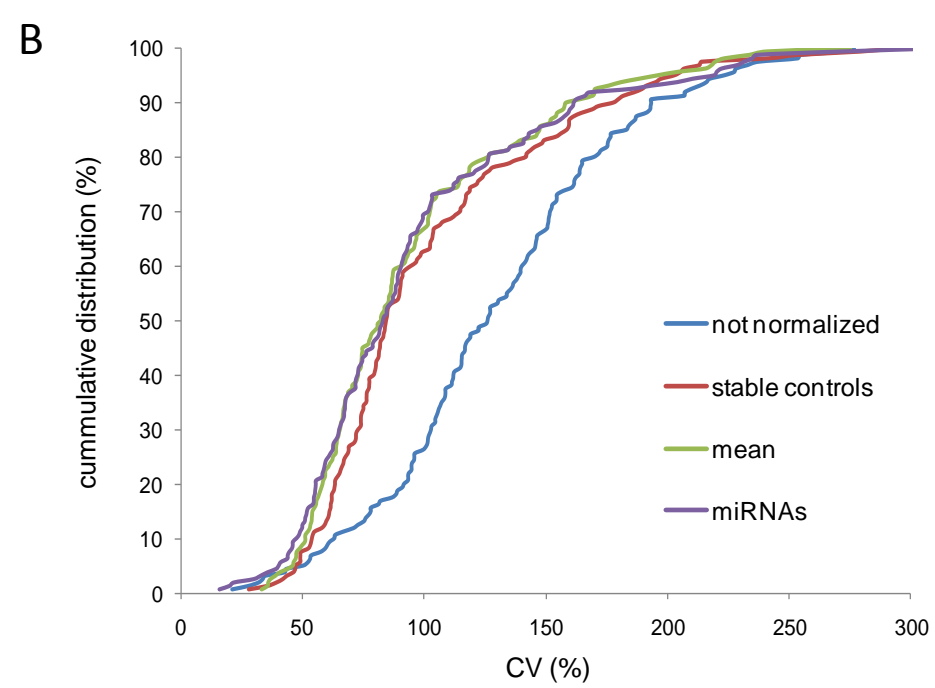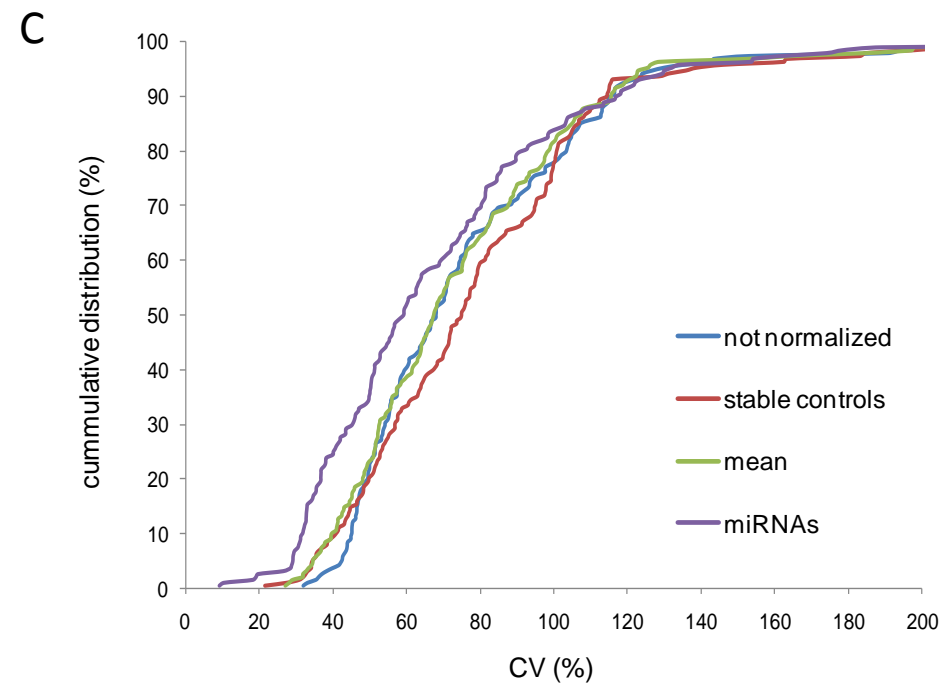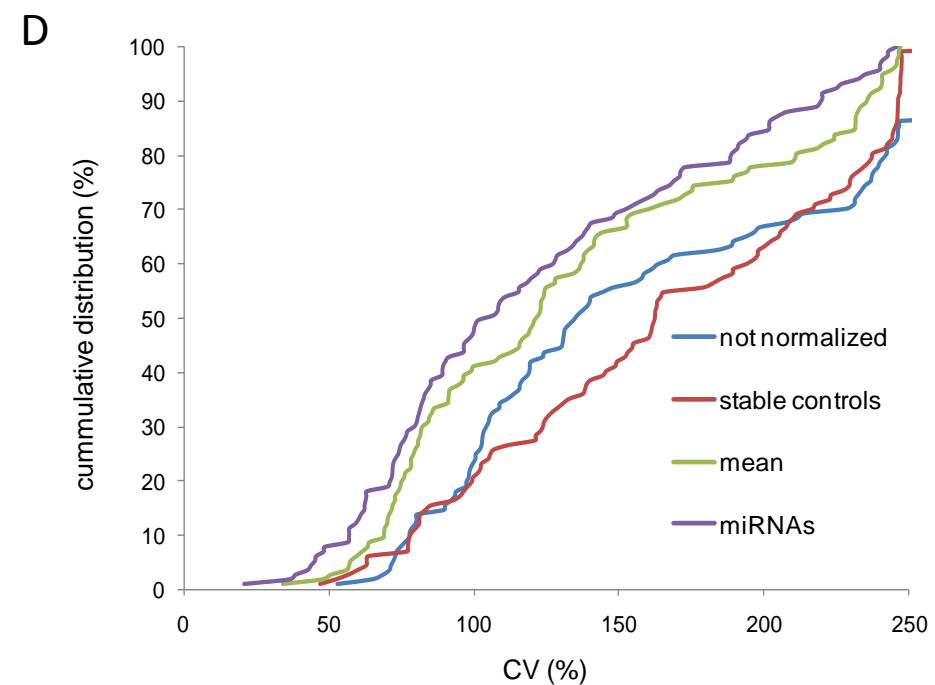

Supplement: Additional data file 3 — The cumulative distribution of miRNA CV-values in (a) the T-ALL sample set, (b) the leukemias with EVI1 overexpression, (c) the normal bone marrow samples and (d) the normal human tissues when no normalization is applied (blue), stable RNA control normalization is applied (red), mean expression value normalization is applied (green) or normalization with miRNAs resembling the mean expression value is applied (purple). Stable small RNA controls for the T-ALL samples: RNU24, RNU44, RNU48, RNU58A, U18 and Z30; for the leukemias with EVI1 overexpression: RNU6B, RNU24 and RNU58A; for the normal bone marrow samples: RNU44, RNU24 and RNU48; for the normal human tissues: RPL21, RNU38B and RNU24. MiRNAs/small RNA controls that resemble the mean expression value are listed in Table 1. [file gb-2009-10-6-r64-S3.pdf]

IMR5/75

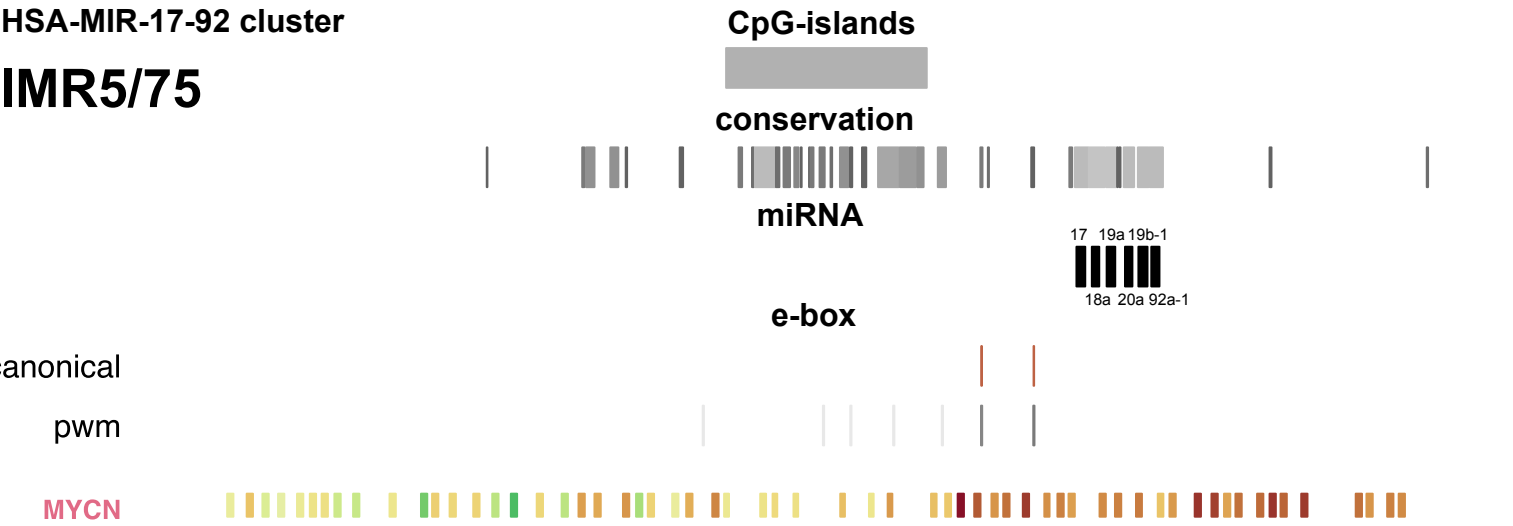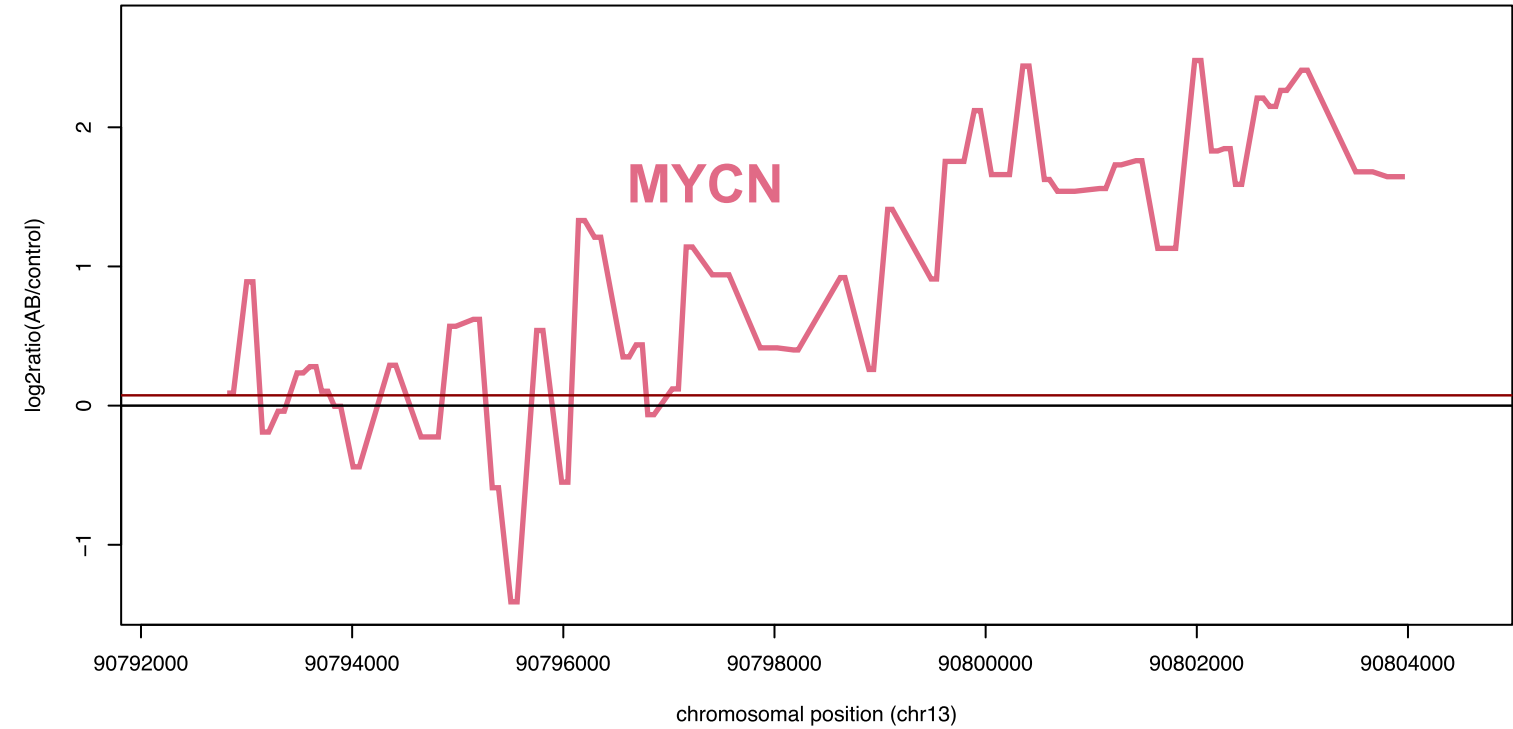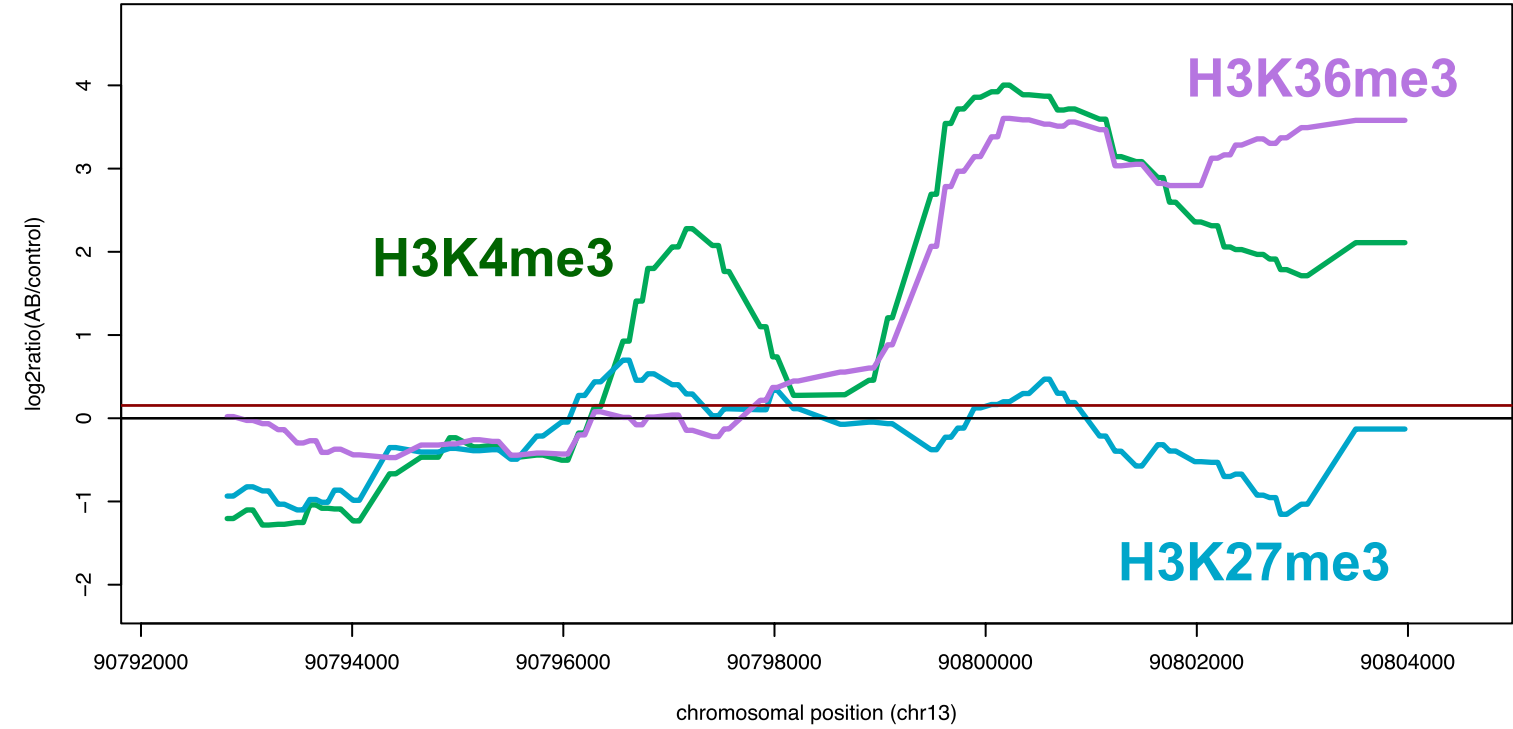

Kelly

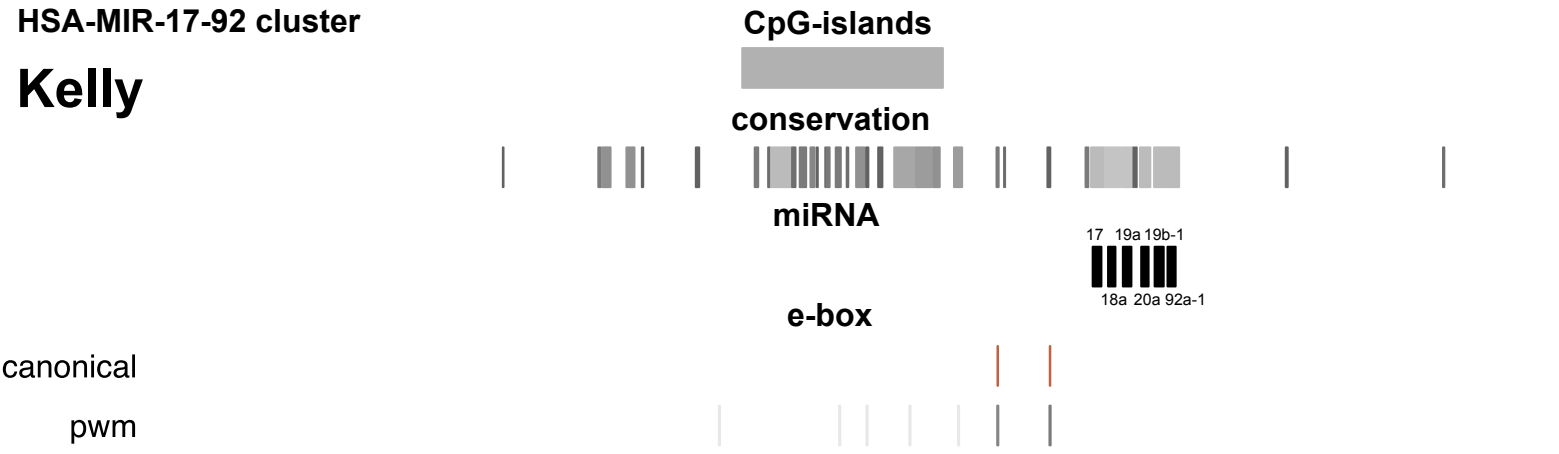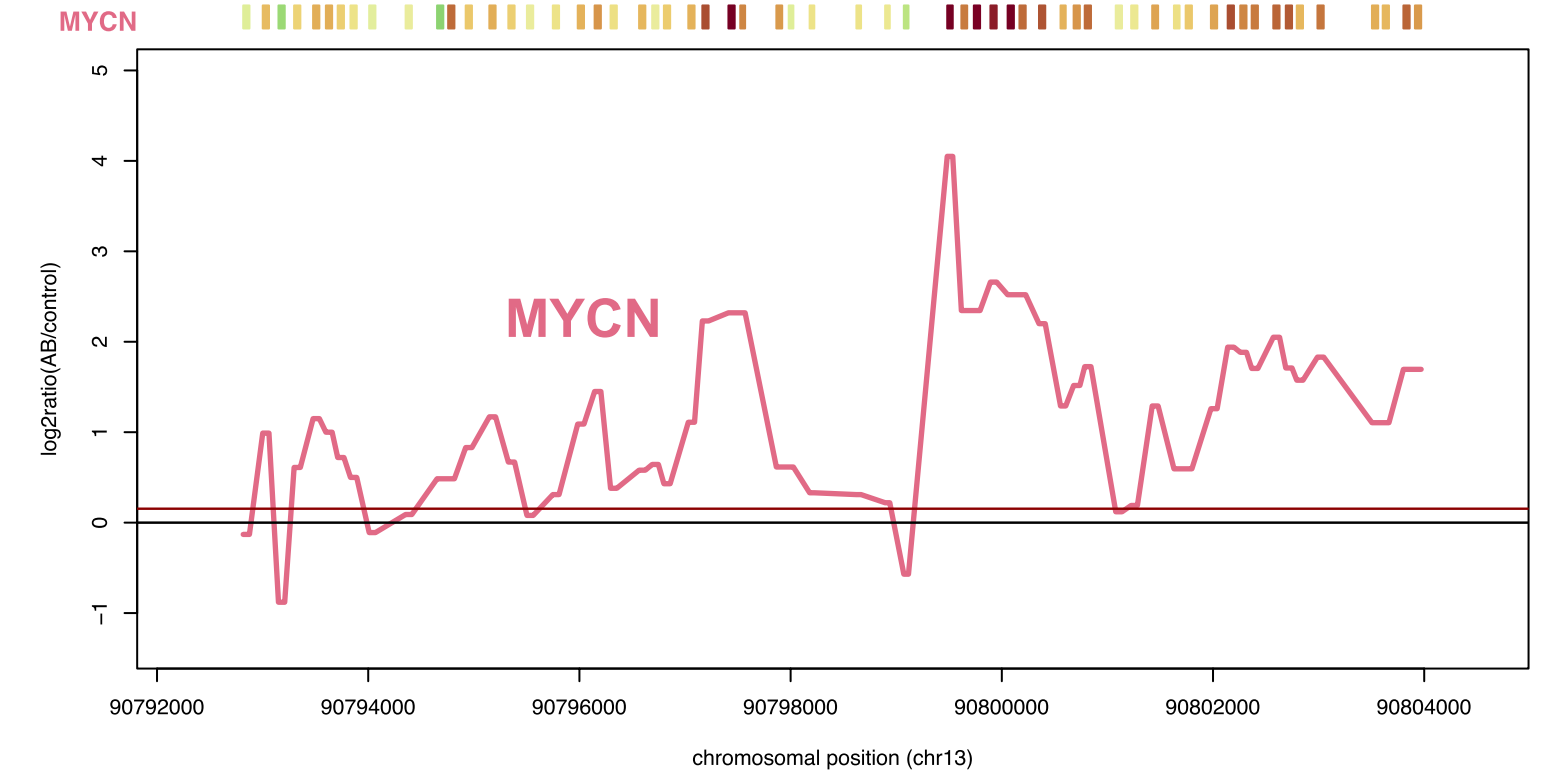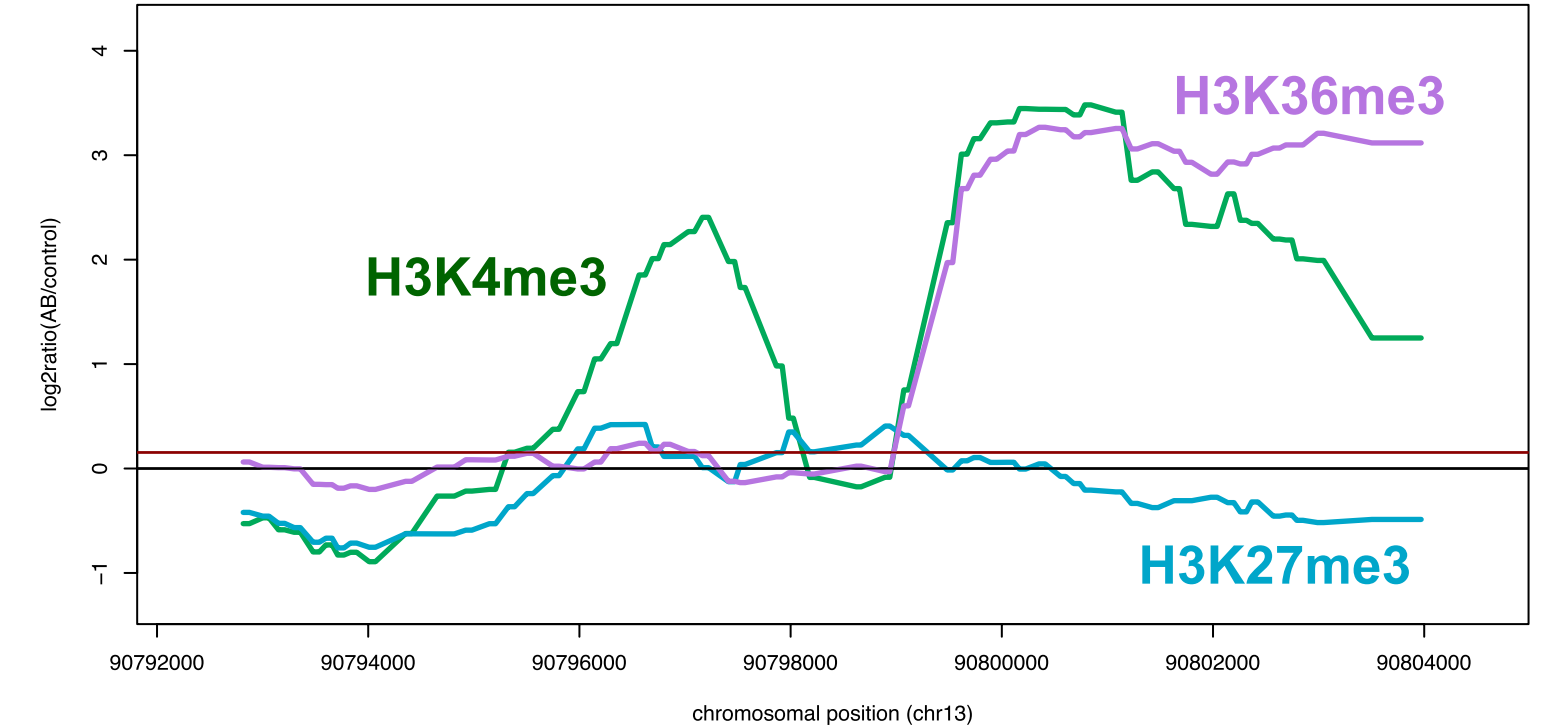

WAC2

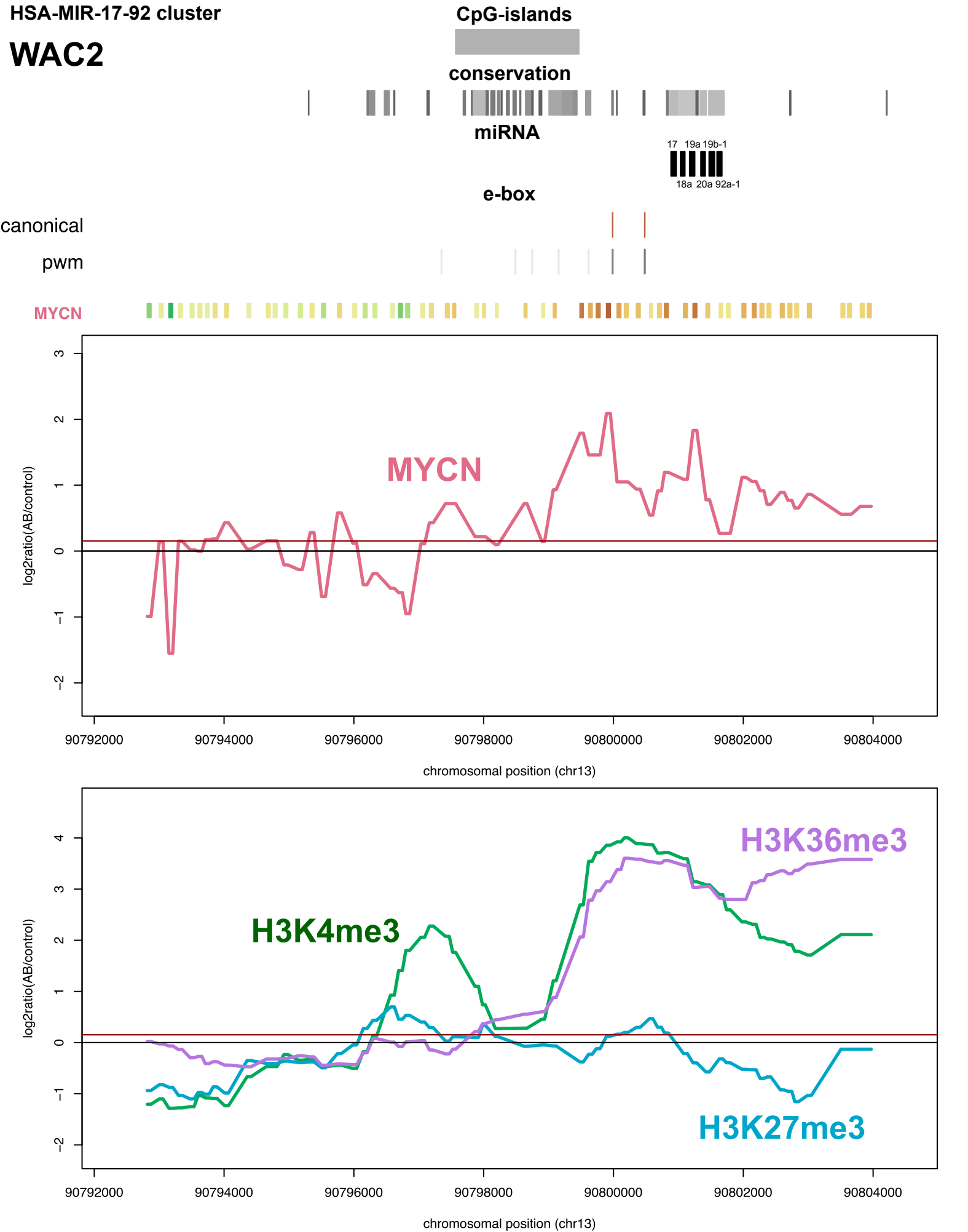

Supplement: Additional data file 4 — ChIP-chip results of the miR-17-92 cluster are given for Kelly, IMR5, and WAC2. Oligonucleotide position is given as bars according to the chromosomal localization. Color coding of the bars represents the log2 ratios MYCN versus input from ChIP-chip experiments, were red means positive and green negative values. Histone marks for active transcription (H3K4me3), repression (H3K27me3) and enlongation (H3K36me3) as measured by ChIP-chip are given together with MYCN binding using the same color coding. miRNA transcript information (miRBase version 11.0), CpG islands, and conservation among 28 species were implemented for the region as given by the respective annotation tracks deposited in the UCSC database (Hg 18, release March 2006). Position of canonical (CACGTG) and non-canonical E-boxes from in silico scanning of the respective sequence is given. Grey coding for results of the positional weight matrix (PWM) scan represents the P-values of the 12 bp MYCN binding motif from the TRANSFAC database. Red line = median log2 ratio MYCN versus input as calculated for each chromosome individually. [file gb-2009-10-6-r64-S4.pdf]

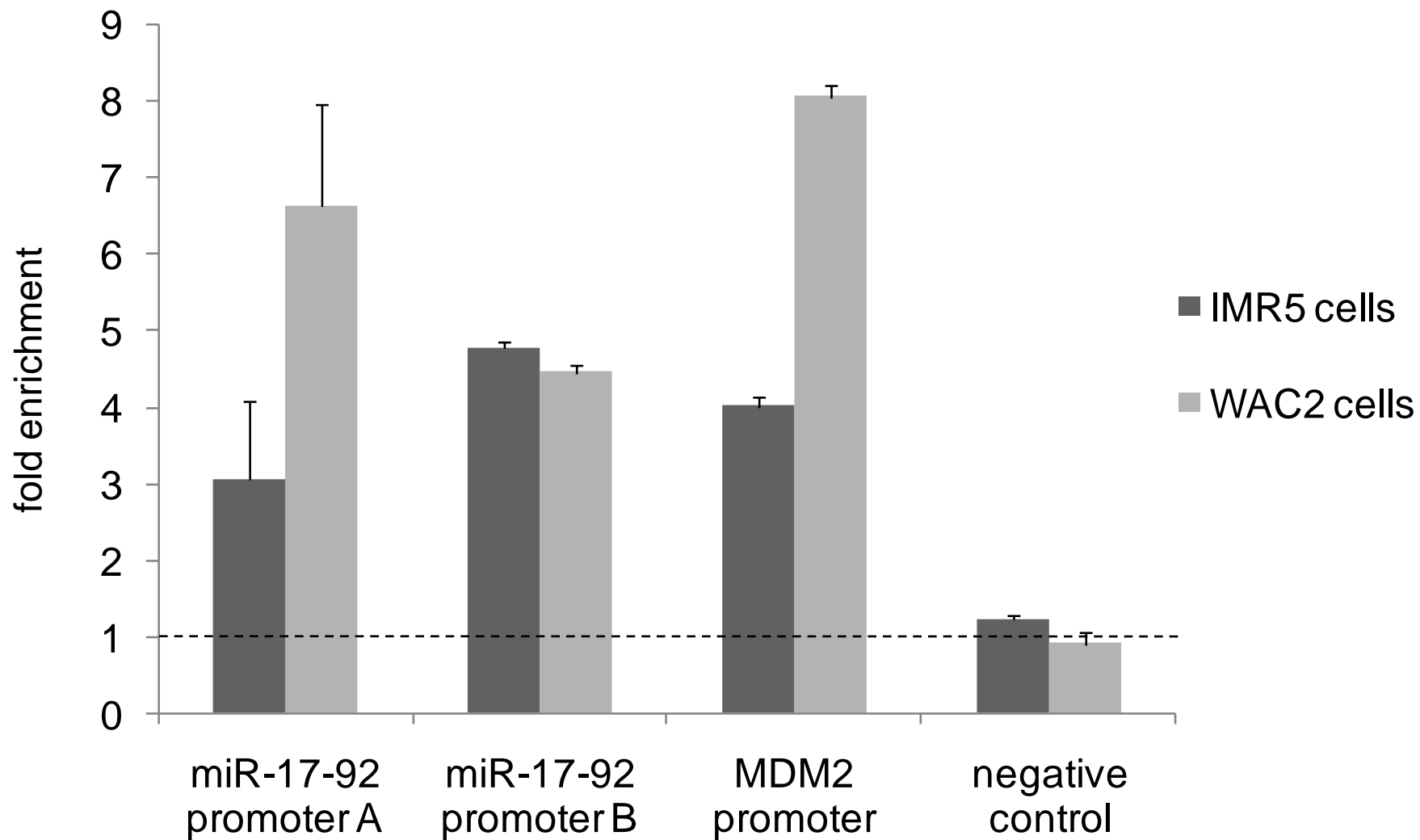

Supplement: Additional data file 5 — Fold enrichment of specific and non-specific genomic regions in the MYCN precipitated samples compared to the input sample as determined by qPCR. MiR-17-92 promoter A and miR-17-92 promoter B are two MYCN specific e-box containing regions in the miR-17-92 promoter. MDM2 promoter is a MYCN specific e-box containing region in the MDM2 promoter and serves as a positive control. The negative control is a non-specific, non e-box containing genomic region. [file gb-2009-10-6-r64-S5.pdf]

IMR5/75

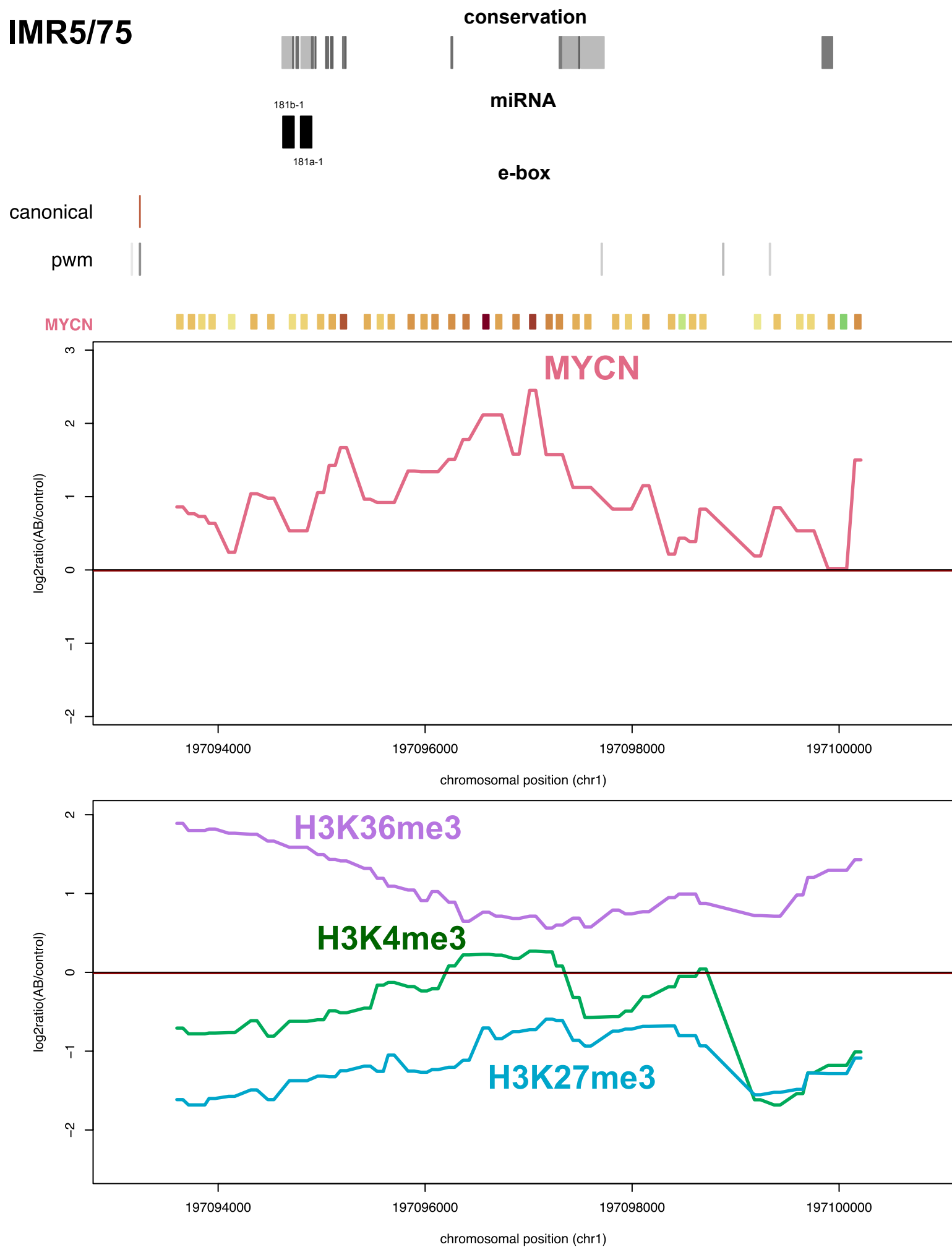

Kelly

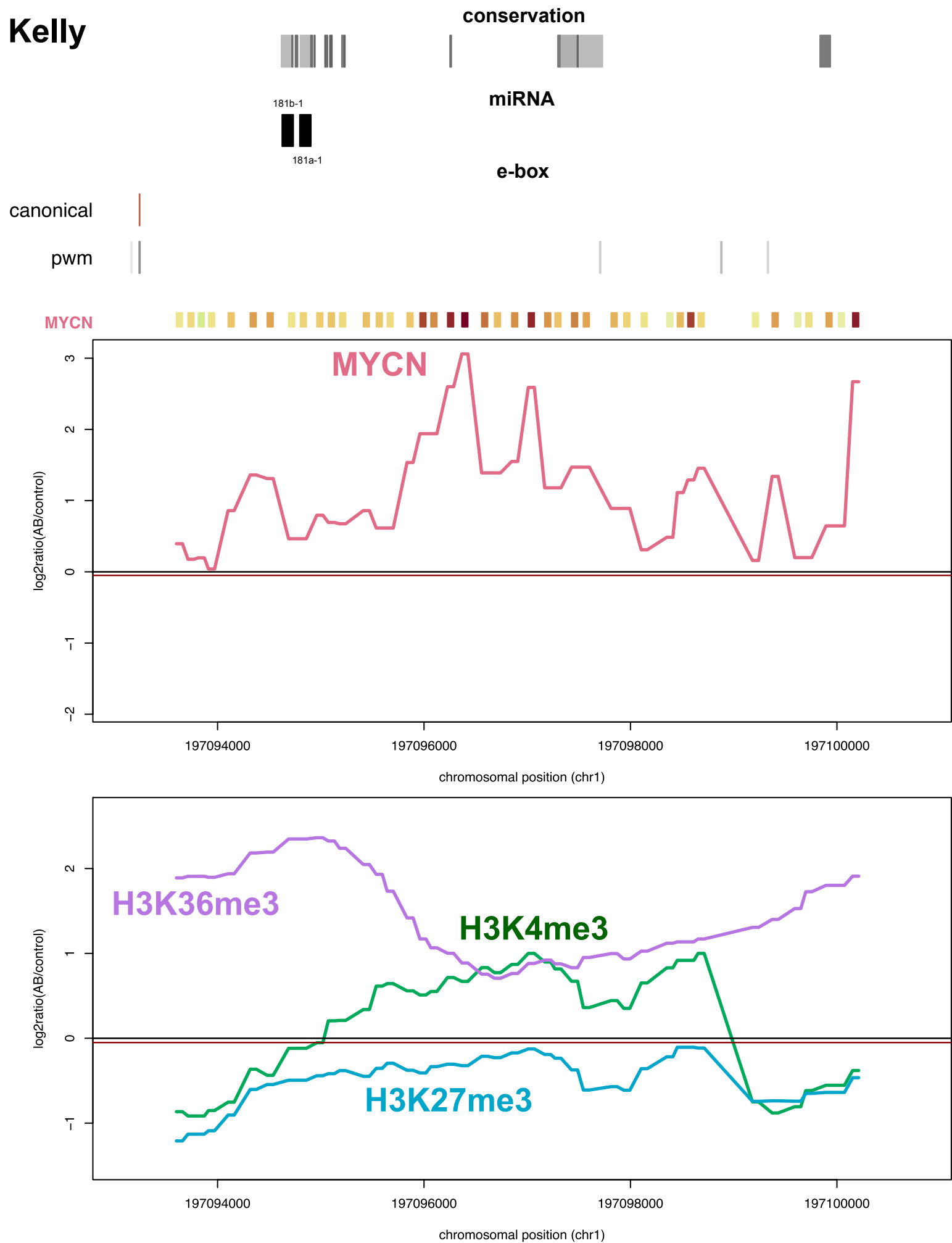

WAC2

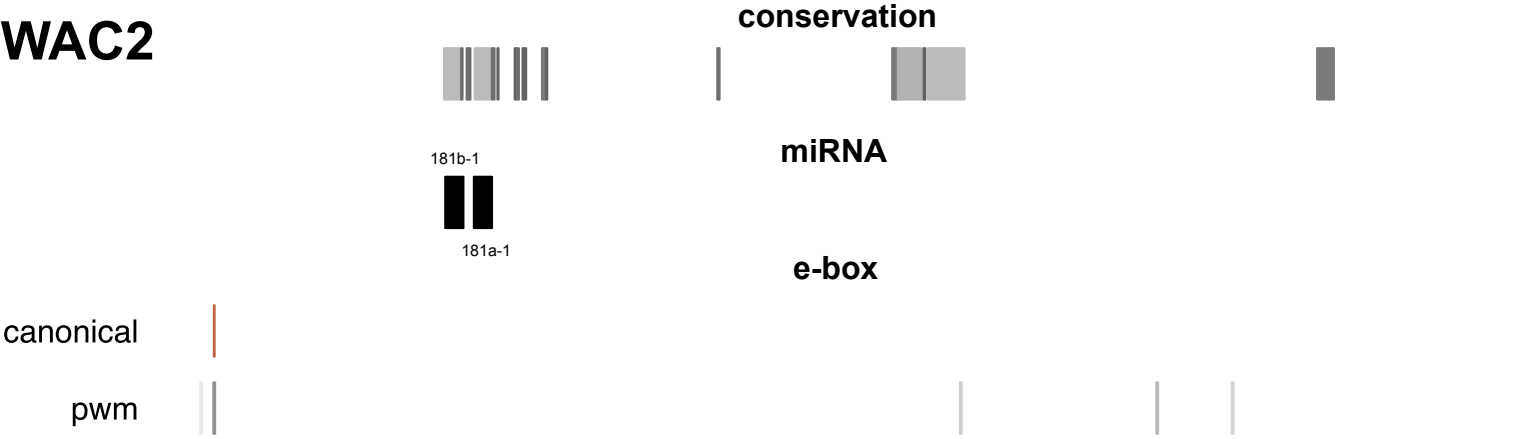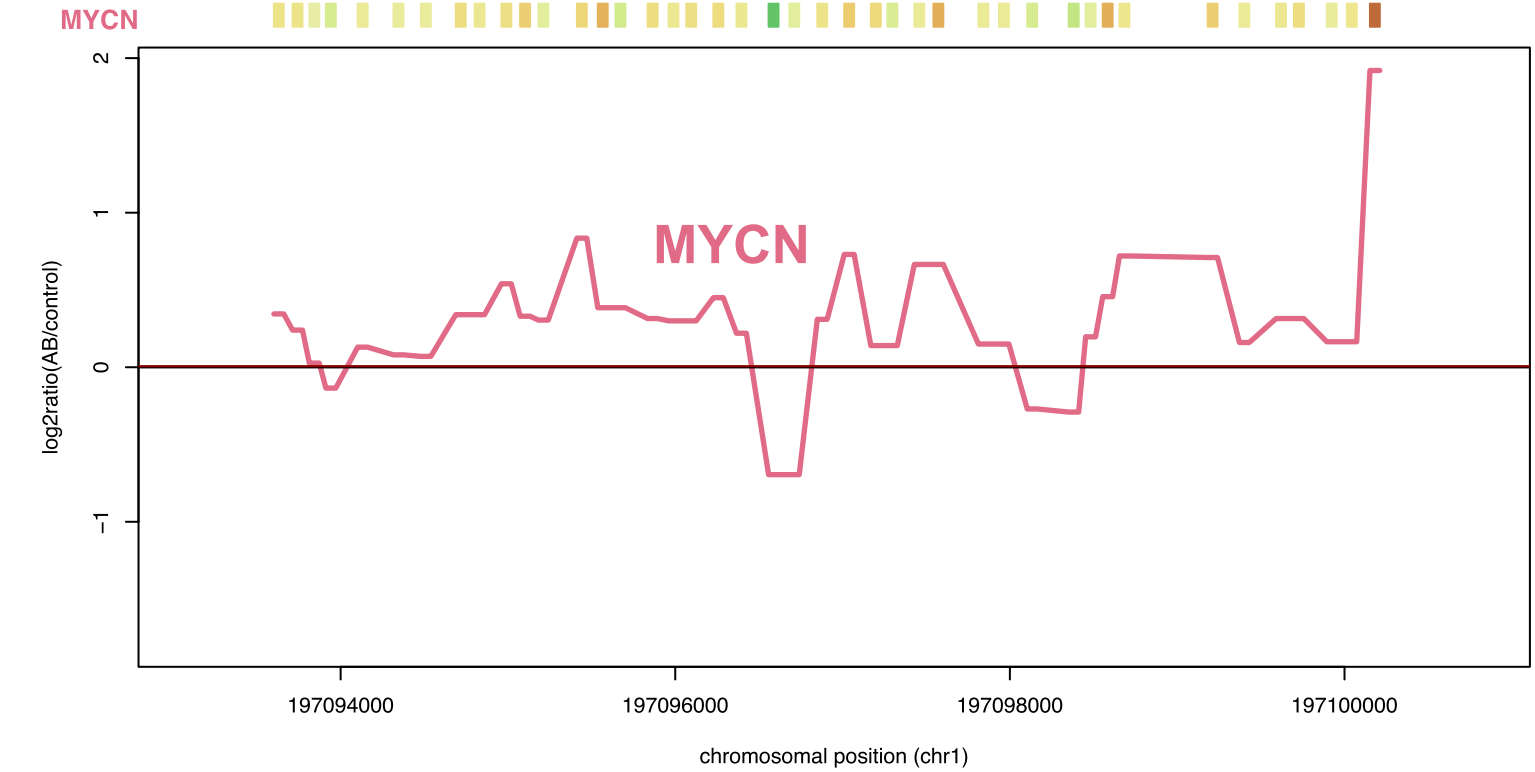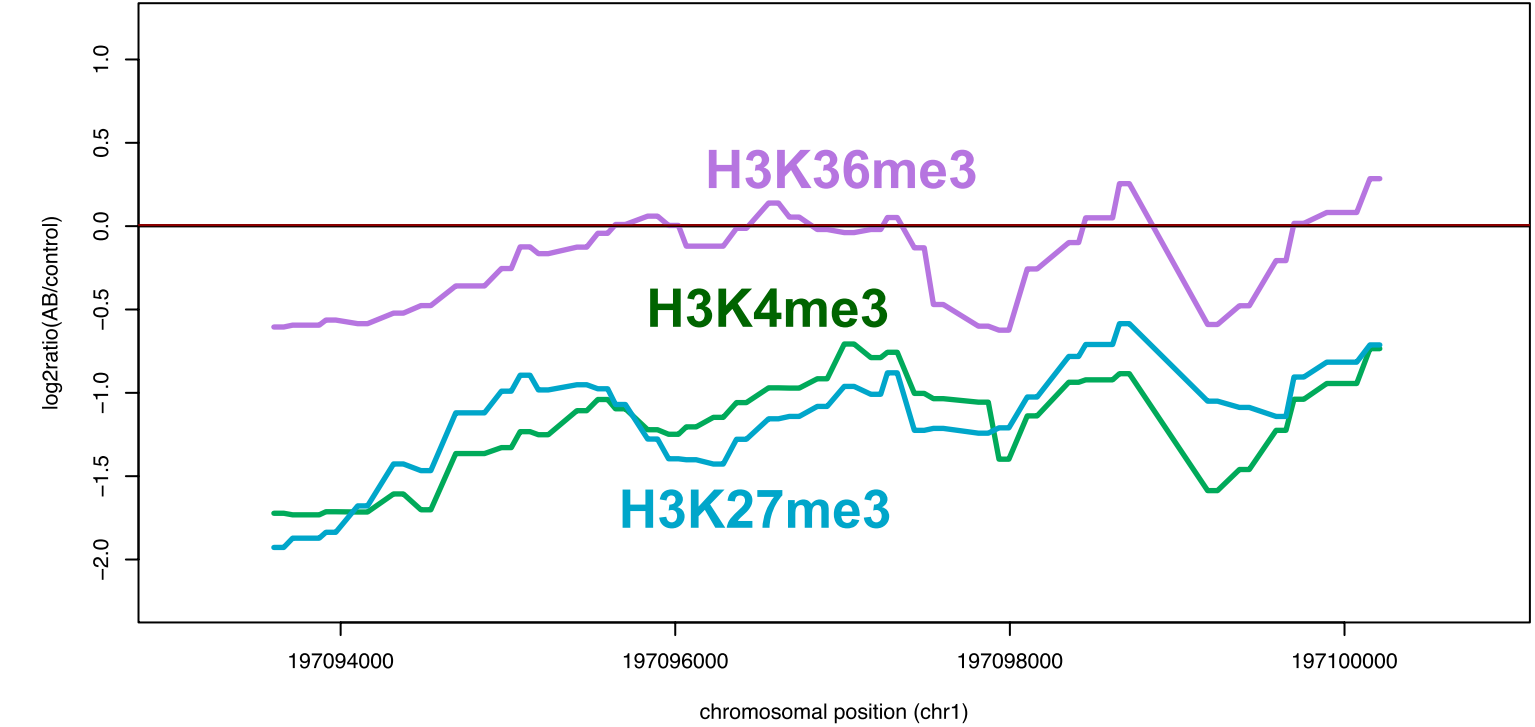

Supplement: Additional data file 6 — ChIP-chip results of the miR-181a-1/miR-181b-1 cluster are given for Kelly, IMR5, and WAC2. Oligonucleotide position is given as bars according to the chromosomal localization. Color coding of the bars represents the log2 ratios MYCN versus input from ChIP-chip experiments, were red means positive and green negative values. Histone marks for active transcription (H3K4me3), repression (H3K27me3) and enlongation (H3K36me3) as measured by ChIP-chip are given together with MYCN binding using the same color coding. miRNA transcript information (miRBase version 11.0), CpG islands, and conservation among 28 species were implemented for the region as given by the respective annotation tracks deposited in the UCSC database (Hg 18, release March 2006). Position of canonical (CACGTG) and non-canonical E-boxes from in silico scanning of the respective sequence is given. Grey coding for results of the positional weight matrix (PWM) scan represents the P-values of the 12 bp MYCN binding motif from the TRANSFAC database. Red line = median log2 ratio MYCN versus input as calculated for each chromosome individually. [file gb-2009-10-6-r64-S6.pdf]

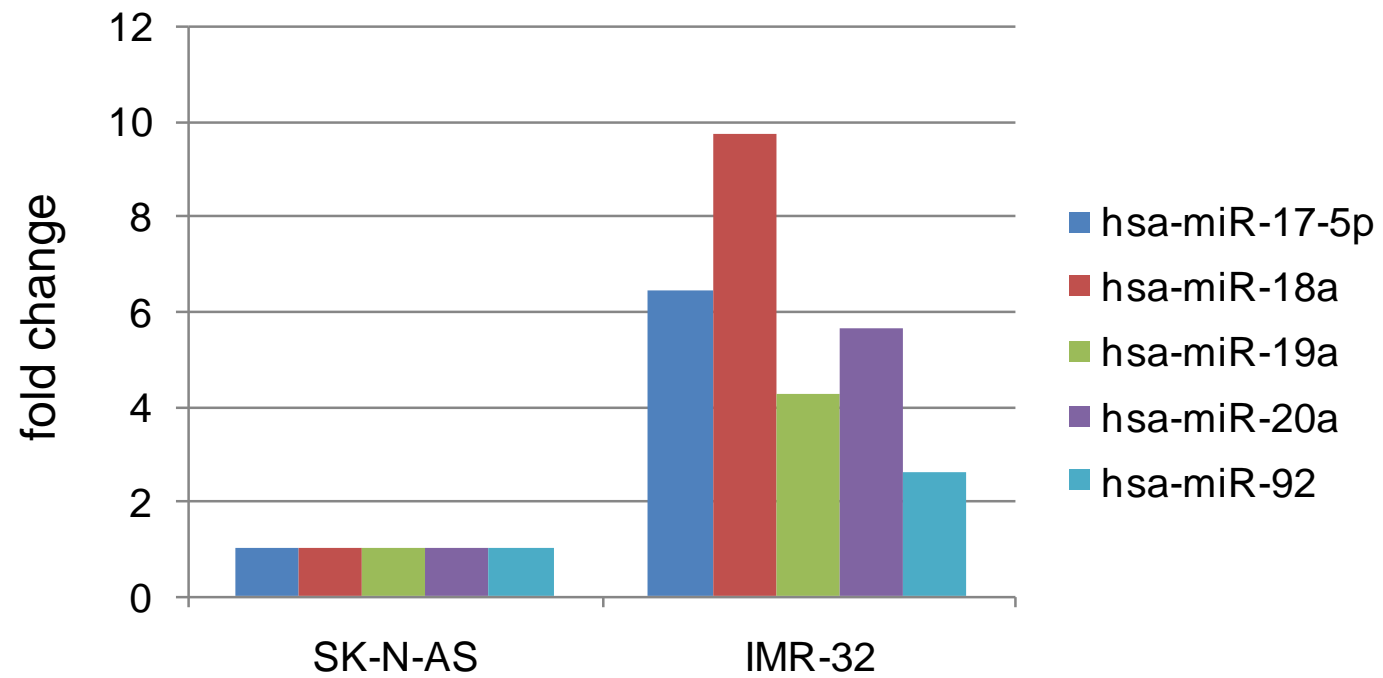

Supplement: Additional data file 7 — Relative expression of miR-17-5p, miR-18a, miR-19a, miR-20a and miR-92a in one MYCN single copy cell line (SK-N-AS) and one MYCN amplified cell line (IMR-32) upon mean expression value normalization. Relative expression values were rescaled to those in SK-N-AS. [file gb-2009-10-6-r64-S7.pdf]

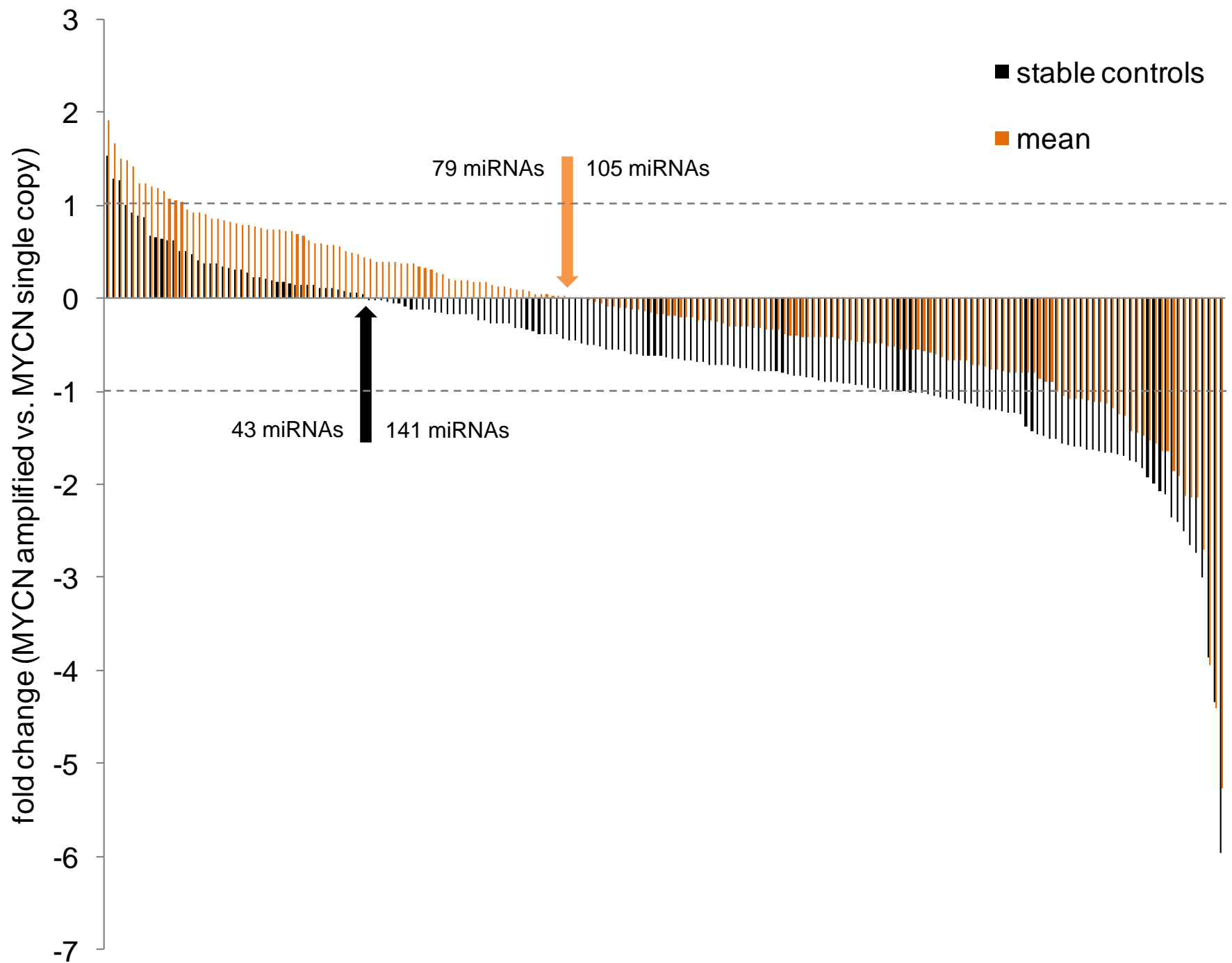

Supplement: Additional data file 8 — Average fold change expression difference of all miRNAs with an expression below the Cq cutoff of 35 PCR cycles in MYCN amplified neuroblastoma samples compared to MYCN single copy neuroblastoma samples. Fold changes were calculated upon stable small RNA control normalization (black) and mean expression value normalization (orange). Plotted fold changes are log2-based and sorted from positive (upregulated in MYCN amplified tumor samples) to negative (downregulated in MYCN amplified tumor samples). Dashed lines represent a two-fold expression difference. Arrows indicate the threshold between up- and downregulated miRNAs for both normalization strategies (the number of up- and downregulated miRNAs is indicated left and right of each arrow, respectively). [file gb-2009-10-6-r64-S8.pdf]

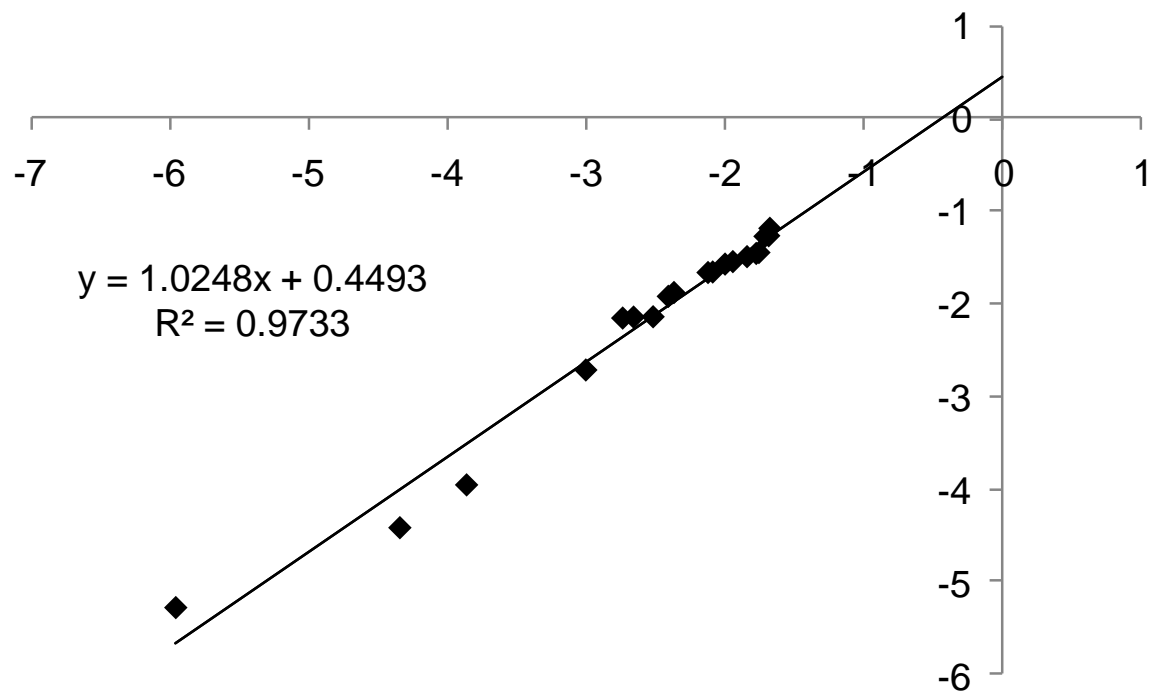

Supplement: Additional data file 9 — Correlation plot showing the average fold change expression difference for the 10% most downregulated miRNAs in MYCN amplified tumors compared to MYCN single copy tumors upon stable small RNA control normalization (x-axis) and mean expression value normalization (y-axis). Both axes are log2-based. The corresponding trend line has a coefficient of determination of 0.973 (R2), a slope approaching 1 and a Y-intercept of 0.449. [file gb-2009-10-6-r64-S9.pdf]

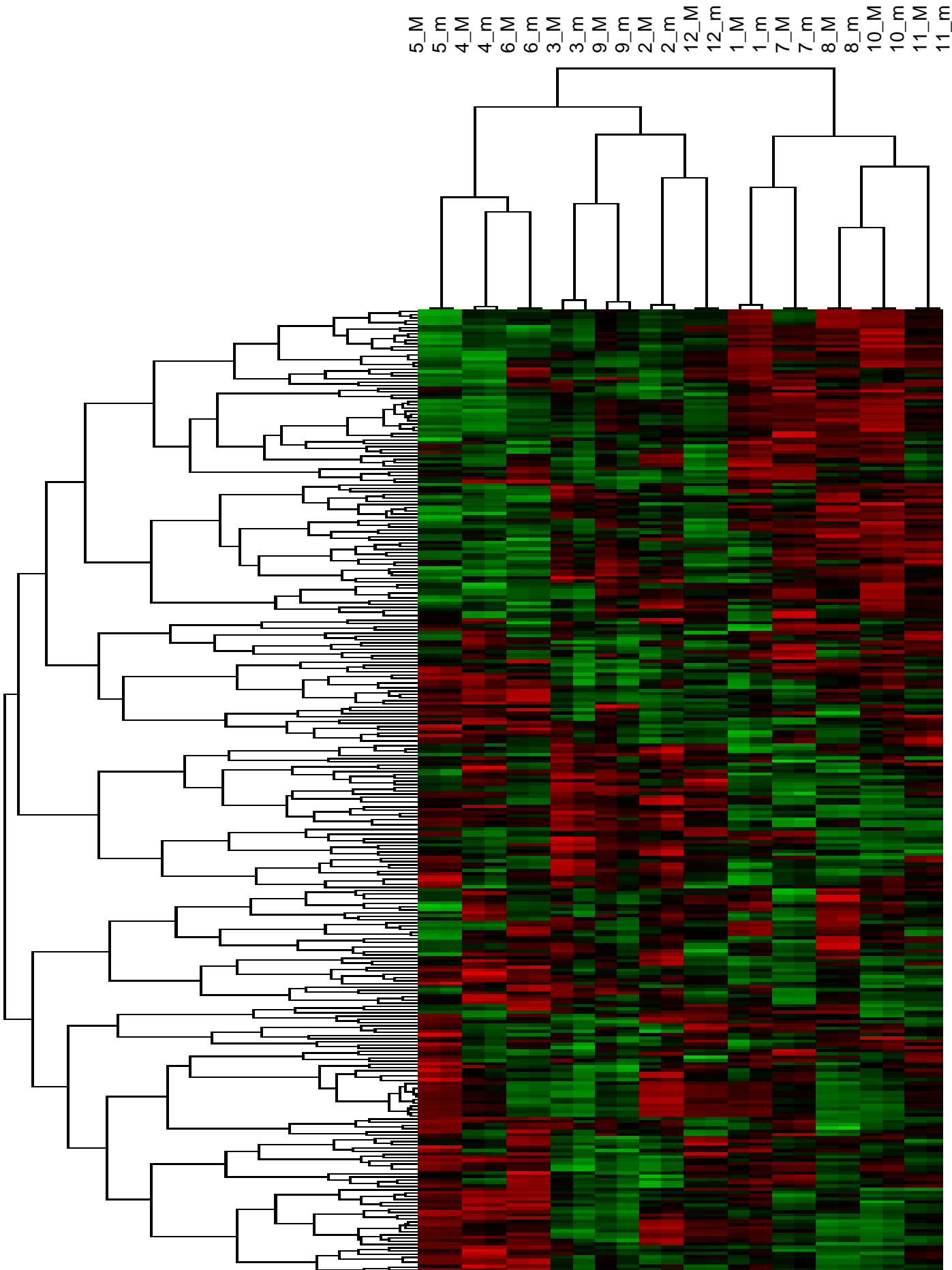

Supplement: Additional data file 10 — Heatmap representing a hierarchical clustering analysis of 24 paired samples based on their miRNA expression profiles. Each sample pair consists of a different neuroblastoma cell line for which the miRNA expression was normalized with the mean expression value or with miRNAs resembling the mean expression value. Cell lines are numbered from 1 to 12. The tag represents the applied normalization strategy (M stands for mean expression value normalization, m for normalization with miRNAs resembling the mean expression value). [file gb-2009-10-6-r64-S10.pdf]
